# CRITID: enhancing CRITIC with advanced independence testing for robust multi-criteria decision-making

**DOI:** 10.1038/s41598-024-75992-z

**Published:** 2024-10-23

**Authors:** Qiang Zhang, Jiahui Fan, Chaobang Gao

**Affiliations:** 1 School of Computer Science, Chengdu University, Chengdu, 610106 China; 2 Key Laboratory of Pattern Recognition and Intelligent Information Processing, Institutions of Higher Education of Sichuan Province, Chengdu University, Chengdu, China; 3 School of Computer and Information Engineering, Jiangxi Normal University, Nanchang, 330022 China; 4 Key Laboratory of Digital Innovation of Tianfu Culture, Sichuan Provincial Department of Culture and Tourism, Chengdu University, Chengdu, China

**Keywords:** CRITID, CRITIC, Independence-zero equivalence property, Independence test, Mathematics and computing, Applied mathematics, Statistics

## Abstract

In multi-criteria decision-making and model evaluation, determining the weight of criteria is crucial. With the rapid development of information technology and the advent of the big data era, the need for complex problem analysis and decision-making has intensified. Traditional CRiteria Importance Through Intercriteria Correlation (CRITIC) methods rely on Pearson correlation, which may not adequately address nonlinearity in some scenarios. This study aims to refine the CRITIC method to better accommodate nonlinear relationships and enhance its robustness. We have developed a novel method named CRiteria Importance Through Intercriteria Dependence (CRITID), which leverages cutting-edge independence testing methods such as distance correlation among others. This approach enhances the assessment of intercriteria relationships. Upon application across diverse data distributions, the CRITID method has demonstrated enhanced rationality and robustness relative to the traditional CRITIC method. These improvements significantly benefit multi-criteria decision-making and model evaluation, providing a more accurate and dependable framework for analyzing complex datasets.

## Introduction

### Literature review on objective weighting methods

The objective weighting method is utilized to ascertain the contributions of multiple criteria or variables towards a defined goal^1^. This technique assigns weights based on rigorous data analysis and statistical methodologies, thereby eliminating reliance on subjective judgments. For instance, within the realm of cluster analysis, the importance of each attribute in the clustering process is ascertained using the objective weighting method. This approach is predicated on the structural properties of the data and mathematical algorithms, as opposed to subjective human assessments. As a result, it ensures that the clustering outcomes are more objective and precise, effectively mitigating potential biases introduced by subjective factors^2^. The objective weighting method is extensively employed across various disciplines, leveraging scientific data analysis techniques to determine the significance of each indicator or variable, thus providing decision-makers with more accurate and reliable decision support.

Currently, commonly utilized objective weighting methods encompass the entropy weight method, standard deviation method, CRITIC method, the Technique for Order of Preference by Similarity to Ideal Solution (TOPSIS)^3^, and Principal Component Analysis (PCA)^4,5^. Each method demonstrates unique performance strengths and weaknesses in various scenarios.

The entropy weight method, based on information entropy, quantifies the degree of discreteness among criteria^6,7^. This method operates under the principle that lower information entropy corresponds to a greater weight assigned to the indicator. Although this method is objective and scientifically grounded, thus reducing subjective bias, it may fail to acknowledge the inherent importance of the indicator.

The Technique for Order of Preference by Similarity to Ideal Solution (TOPSIS) method evaluates the quality of samples by approximating an ideal solution^3,7^. This method is intuitive and simple to comprehend. However, it encounters limitations in scenarios where criteria exhibit slight or sudden changes, and it may introduce a degree of subjective bias.

Principal Component Analysis (PCA) reduces data dimensionality and extracts salient features by transforming the original criteria into a set of uncorrelated principal components, weighting them according to their contributions^5^. This method effectively eliminates data correlation and reduces redundancy and complexity. However, PCA necessitates determining the number of principal components and presupposes a linear data distribution, which may introduce potential subjective judgments and risk ineffective dimensionality reduction.

The CRITIC method, proposed by Diakoulaki, Mavrotas, and Papayannakis in 1995^8^, is employed to ascertain the weights of multiple criteria in decision-making processes^9–11^. This method evaluates the importance of each indicator by analyzing both the correlation among criteria and the variability of the criteria themselves. Compared to other objective weighting methods, the CRITIC method offers several advantages. It accounts for the correlation between criteria, emphasizing not only the variation of each indicator but also the potential interrelations among different criteria. By analyzing these correlations, the CRITIC method can more accurately assess the impact of each indicator on the final decision, thereby enhancing the accuracy and reliability of decision-making. This method derives weights directly from the data’s inherent correlation information, making it highly objective and scientifically valid. In contrast, other methods such as TOPSIS and PCA may involve subjective parameter settings or require statistical handling, introducing a degree of subjectivity. The CRITIC method is notable for its robust applicability and flexibility, suitable for a wide range of decision problems and compatible with any number of criteria. Whether addressing simple decision-making scenarios or complex multi-criteria decision-making issues, the CRITIC method provides an effective solution for weight determination. By analyzing the correlation among criteria and their variability, the CRITIC method demonstrates substantial advantages in accuracy, objectivity, and applicability, offering decision-makers a scientific and reliable approach to weight determination that significantly enhances the quality and efficiency of decision-making.

Numerous methods have been developed to augment the CRITIC method with fuzzy set theory, thereby providing more reliable and nuanced decision support in uncertain environments. Significant contributions in this domain can be found in^12–21^. The incorporation of fuzzy logic into the CRITIC-REGIME decision-making method offers a sophisticated approach for evaluating and selecting alternatives under conditions of uncertainty^12^. By leveraging fuzzy logic methodologies, these papers deliver comprehensive tools for decision-makers, significantly improving decision quality in complex scenarios where multiple criteria must be considered.

The CRITIC method has been extensively utilized across various fields. Yalcin et al.^22^ employed the CRITIC method to perform a multi-criteria performance analysis of initial public offerings (IPOs), providing investors with a structured guide to making more informed and profitable investment decisions. Furthermore, Ayşegül Tuş and Esra Aytaç Adalı^23^ implemented a hybrid approach combining the CRITIC method with the WASPAS method to address the problem of selecting attendance software in private hospitals.

### Study motivation

Although the CRITIC method has been widely used across various fields, it exhibits some limitations. The Pearson correlation used in the CRITIC method can only measure linear correlation. The need to assess true dependence or independence among complex objects arises in various domains, including medical imaging, computational biology, and computer vision. There exist numerous statistical methods designed to test dependence beyond linear correlation. For instance, Gábor J. Székely, Maria L. Rizzo, and Nail K. Bakirov introduced the concept of distance covariance in 2007^24^, which measures the distance between the joint characteristic function of two random vectors and the product of their respective marginal characteristic functions. The definition of the distance correlation coefficient is analogous to that of the Pearson correlation coefficient, involving the division of the distance covariance by the product of the respective distance variances under the root sign. Unlike the Pearson correlation, a distance correlation coefficient of zero signifies complete independence between two random vectors, whereas a zero Pearson correlation coefficient merely indicates the absence of a linear relationship. The distance correlation coefficient is capable of capturing non-linear relationships, an ability not possessed by the Pearson correlation coefficient. Additional methods can be found in^25–28^.

### Objectives and contributions

The primary contributions of this study are summarized as follows:

- We introduce a novel method, named CRiteria Importance Through Intercriteria Dependence (CRITID), which leverages advanced independence tests such as distance correlation^24^, ball covariance^26^, and projection correlation^25^. This approach addresses the limitations of Pearson correlation used in CRITIC, which is restricted to measuring linear relationships.
- Through both simulation and analysis of real data, the proposed CRITID method demonstrates superior efficiency, yielding more accurate results with lower mean squared error (MSE) compared to existing methods.
- Sensitivity analysis confirms that CRITID is more robust than the traditional CRITIC method, enhancing reliability in diverse analytical contexts.
- The applicability of the CRITID method extends across a wide range of real-world data scenarios, proving its versatility and effectiveness.

### Structure of the article

The remainder of this article is organized as follows. Section 2 introduces the CRITID method, including its foundational definition and the development of the CRITID-Dcor method. This section also discusses the existing challenges within the CRITIC framework and explicates concepts like distance covariance and distance correlation coefficient. Section 3 evaluates the CRITID-Dcor method’s efficacy and robustness using various simulated data distributions. Section 4 applies the CRITID-Dcor method to real datasets, including data on Parkinson’s disease and smart education evaluation, to assess its practical effectiveness and robustness in real-world scenarios. Section 5 summarizes the findings, discusses the limitations of the CRITID-Dcor method, and outlines directions for future research.

## CRITID method

### CRITID method definition

The CRITIC method systematically quantifies the objective weight between criteria by calculating the product of the contrast strength within the criteria and the conflict between them. Prior to employing the CRITIC method for weight calculation of an indicator, it is essential to normalize the indicator to mitigate the effects of its dimensional and value range variances. The relative importance and weight of each indicator are defined as follows.

#### Definition 1

(CRITIC method) Suppose that there are *m* criteria. Let $a_k$ denote the standard deviation of the *k*-th criterion, and $r_{ik}$ represent the Pearson correlation coefficient between criteria *i* and *k*. Then, the importance of the *k*-th criterion is defined by the equation:

$$\begin{aligned} C_k = a_k \left[ \sum _{i=1}^m (1 - r_{ik})\right] , \quad k = 1, 2, \ldots , m. \end{aligned}$$

The weight of the *k*-th criterion is given by:

$$\begin{aligned} W'_k = \frac{C_k}{\sum _{b=1}^m C_b}. \end{aligned}$$

In the provided definition, the term $\sum _{i=1}^m(1-r_{ik})$ represents the conflict or independence between the *k*-th criterion and other criteria. According to Definition 1, if the correlation between criteria is positive, $\sum _{i=1}^m\left( 1-r_{ik}\right)$ should be interpreted as the degree of independence between criteria. Conversely, if the correlation is negative, it should be seen as the conflict between criteria.

The Pearson correlation coefficient, which measures only linear correlations, is insufficient for capturing the complete independence between criteria when correlations are positive. In such cases, replacing the Pearson correlation coefficient with a metric capable of detecting non-linear relationships would more accurately describe the independence of criteria. For negative correlations, the CRITIC method assigns higher weights to criteria with stronger negative correlations, potentially leading to data redundancy. For example, two criteria with perfect negative correlation would both receive disproportionately high weights, effectively double-counting their contributions and diminishing the relative importance of other criteria. This can lead to an unfair weighting distribution.

To address this issue, incorporating the absolute value of the correlation coefficient could mitigate the problem of high data redundancy, irrespective of whether the correlation is positive or negative. Nevertheless, since the Pearson correlation coefficient is limited to linear relationships, it remains advisable to consider an alternative index capable of capturing nonlinear relationships to more effectively resolve these issues.

#### Definition 2

(*CRITID Method*) Consider a set of *m* criteria. Let $a_k$ represent the standard deviation of the *k*-th criterion, and let $s_{ik}$ denote a dependence measure coefficient between criterion *i* and criterion *k*. The significance of the *k*-th criterion is quantified by the equation:

1 $$\begin{aligned} D_k = a_k \left[ \sum _{i=1}^m (1 - s_{ik})\right] , \quad k = 1, 2, \ldots , m. \end{aligned}$$

The weight assigned to the *k*-th criterion is then defined as:

2 $$\begin{aligned} W_k = \frac{D_k}{\sum _{b=1}^m D_b}. \end{aligned}$$

This weighting method dynamically adjusts the influence of each criterion based on its variance and the level of independence from other criteria.

#### Remark 1

The dependence measure coefficient, $s_{ik}$, is selected based on its adherence to the Independence-zero equivalence property, as described by^26^. This property ensures that the test statistic is zero if and only if the two random vectors are independent, thereby facilitating the detection of a wide range of dependency types, including nonlinear correlations. Methods satisfying this property include, but are not limited to, those detailed in^24,26–28^, which underscore the robustness and versatility of these statistical tools in capturing diverse forms of interdependencies among variables.

**Figure 1:**
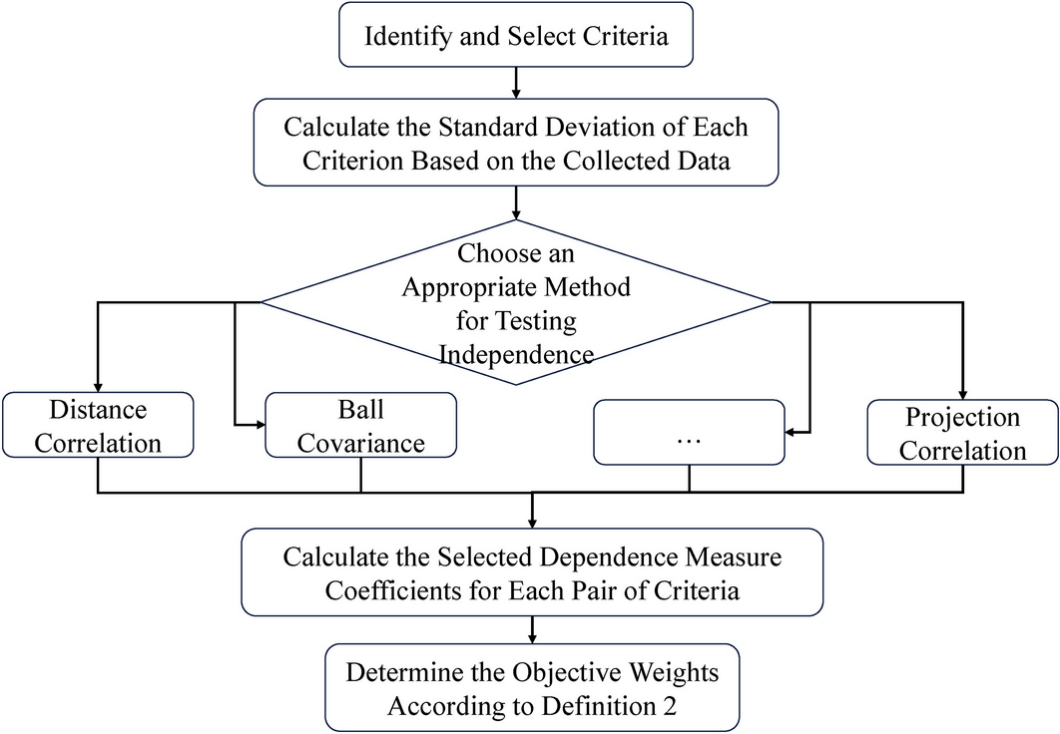
Flowchart illustrating the sequential steps of the CRITID methodology.

The flowchart illustrating the sequential steps of the CRITID methodology is presented in Fig. 1. In the subsequent section, we will utilize the distance correlation method, as described in^24^, to demonstrate the efficacy of the CRITID approach.

### Distance correlation

Distance covariance quantifies the divergence between the product of the joint characteristic function of two variables, *X* and *Y*, and the product of their marginal characteristic functions.

#### Definition 3

(*Distance covariance*) Assume that the random vectors $X \in \mathbb {R}^p$ and $Y \in \mathbb {R}^q$ have finite first-order moments. The distance covariance, denoted as *V*(*X*, *Y*), is defined by the square root of the following equation:

3 $$\begin{aligned} \begin{aligned} V^2(X,Y)&= \Vert f_{X,Y}(t,s) - f_X(t)f_Y(s)\Vert ^2 \\&= \frac{1}{c_p c_q} \int _{\mathbb {R}^{p+q}} \frac{|f_{X,Y}(t,s) - f_X(t) f_Y(s)|^2}{|t|_p^{1+p} |s|_q^{1+q}} \, dt \, ds, \end{aligned} \end{aligned}$$

where $f_X$ and $f_Y$ are the characteristic functions of *X* and *Y*, respectively, and $f_{X,Y}$ is the joint characteristic function of *X* and *Y*. The normalization constants $c_p$ and $c_q$ are defined as $c_p = \frac{\pi ^{(1+p)/2}}{\Gamma ((1+p)/2)}$ and $c_q = \frac{\pi ^{(1+q)/2}}{\Gamma ((1+q)/2)}$, with $\Gamma (\cdot )$ being the complete gamma function, $\Gamma (z) = \int _0^\infty t^{z-1} e^{-t} \, dt$.

From Equation (3), it is evident that distance covariance represents the norm of the difference between the product of the joint characteristic functions of two variables and their respective marginal characteristic functions. Following the definition of distance covariance, a special case arises where the distance variance is equivalent to the distance covariance, specifically when considering the same variable. This is formally defined as:

$$\begin{aligned} V^2(X) = V^2(X, X) = \Vert f_{X,X}(t,s) - f_X(t)f_X(s)\Vert ^2. \end{aligned}$$

The distance correlation coefficient is derived from the distance covariance and distance variance. The formal definition is provided below.

#### Definition 4

(*Distance correlation coefficient*) For random vectors *X* and *Y* with finite first-order moments, the distance correlation coefficient *R*(*X*, *Y*) between them is a non-negative number defined as:

$$\begin{aligned} R^2(X,Y) = {\left\{ \begin{array}{ll} \frac{V^2(X,Y)}{\sqrt{V^2(X) V^2(Y)}}, & \text {if } V^2(X)V^2(Y) > 0,\\ 0, & \text {if } V^2(X)V^2(Y) = 0. \end{array}\right. } \end{aligned}$$

Like the Pearson correlation coefficient, the distance correlation coefficient not only bears a similar formulation but also exhibits analogous properties. These properties are detailed in Theorem 3 as presented in^24^.

#### Theorem 1

(Theorem 3 in^24^) *Assume that random vectors*$X \in \mathbb {R}^p$*and*$Y \in \mathbb {R}^q$. *They exhibit the following properties:*

1. If $E\left( |X|_p + |Y|_q\right) < \infty$, *then*$0 \le R(X,Y) \le 1$, *and*$R(X,Y) = 0$*if and only if* *X* *and* *Y* *are* *independent.*
2. If $R(X,Y) = 1$, *then there exists a vector*$a \in \mathbb {R}^q$, *a non-zero real number* *b*, *and an orthogonal matrix* *C* *such that*$Y = a + bXC$.

It can be seen from the Theorem 1 that, unlike the Pearson correlation coefficient, when the distance correlation coefficient $R(X,Y) = 0$, *X* and *Y* are independent. However, when $R(X,Y) = 1$, a relationship similar to that observed with the Pearson correlation coefficient when its absolute value is 1 exists. Specifically, there exists a linear transformation such that $Y = aX + b$, analogous to the condition $Y = a + bXC$ required by the distance correlation. This signifies a deterministic linear relationship between *X* and *Y*, but with the distinction that for distance correlation, *C* must be an orthogonal matrix, indicating more specific constraints than those imposed by Pearson correlation.

To sum up, the distance correlation coefficient is a generalization of the correlation coefficient. It not only captures linear relationships between variables but also nonlinear ones. Moreover, the distance correlation coefficient offers several advantages over the Pearson correlation coefficient:

1. The distance correlation coefficient is invariant to scale transformations of the variable. That is, when a variable is multiplied by a constant or shifted by a constant, the distance correlation coefficient remains unchanged. In contrast, the Pearson correlation coefficient can lead to misleading interpretations, especially in the presence of outliers.
2. The distance correlation coefficient can measure both linear and nonlinear dependencies. This is a significant extension over the Pearson correlation coefficient, which is limited to measuring only linear dependence.
3. The distance correlation coefficient is robust to outliers in the data. Outliers have minimal impact on the calculation of the distance correlation coefficient, making it a more reliable measure in the presence of extreme observations.
4. The distance correlation coefficient exhibits ideal statistical properties, including consistency. As the sample size increases, the distance correlation coefficient converges to its population value, providing reliable estimates even with limited data.

The definition and advantages of the distance correlation coefficient have been discussed above. In practical applications, it is essential to calculate the distance correlation coefficient between two samples, defined as follows.

### CRITID-Dcor method

#### Definition 5

(*Empirical distance covariance*) For the observed random sample $(X, Y) = \{(X_{k}, Y_{k}) : k = 1, 2, 3, \ldots , n\}$, with $X \in \mathbb {R}^p$ and $Y \in \mathbb {R}^q$ following a joint distribution, define the following:

$$\begin{aligned} a_{kl}&= |X_k - X_l|_p,&\overline{a}_{k \cdot }&= \frac{1}{n} \sum _{l=1}^n a_{kl}, \\ \overline{a}_{\cdot l}&= \frac{1}{n} \sum _{k=1}^n a_{kl},&\overline{a}_{..}&= \frac{1}{n^2} \sum _{k,l=1}^n a_{kl}, \\ A_{kl}&= a_{kl} - \overline{a}_{k \cdot } - \overline{a}_{\cdot l} + \overline{a}_{..}, \end{aligned}$$

where $k, l = 1, 2, 3, \ldots , n$. Similarly, define $b_{kl}$ and $B_{kl}$ for *Y* following the same procedure. Then, the sample distance covariance $V_n(X,Y)$ is defined as the square root of:

$$\begin{aligned} V_n^2(X,Y) = \frac{1}{n^2} \sum _{k,l=1}^n A_{kl} B_{kl}. \end{aligned}$$

Similarly, the sample distance variance for *X* is defined as:

$$\begin{aligned} V_n^2(X) = V_n^2(X, X) = \frac{1}{n^2} \sum _{k,l=1}^n A_{kl}^2. \end{aligned}$$

#### Definition 6

(*Empirical distance correlation coefficient*) The sample distance correlation coefficient, denoted as $R_n(X,Y)$, is defined as the square root of the following equation:

$$\begin{aligned} R_n^2(X,Y) = {\left\{ \begin{array}{ll} \frac{V_n^2(X,Y)}{\sqrt{V_n^2(X) V_n^2(Y)}}, & \text {if } V_n^2(X) V_n^2(Y) > 0,\\ 0, & \text {if } V_n^2(X) V_n^2(Y) = 0. \end{array}\right. } \end{aligned}$$

Given the discussed advantages of the distance correlation coefficient over the Pearson correlation coefficient, it is rational to integrate this measure into the original CRITIC framework. This integration leads to the development of the CRITID-Dcor method, which is defined as follows.

#### Definition 7

(*CRITID-Dcor*) Assume there are *m* criteria $X_1, X_2, \ldots , X_m$. Let $\textrm{dcov}_{ik} = V_n(X_i, X_k)$ represent the distance covariance between criteria *i* and *k*. The importance of the *k*-th criterion is calculated using the formula:

$$\begin{aligned} D_k = a_k \left[ \sum _{i=1}^m \left( 1 - \frac{\textrm{dcov}_{ik}}{\sqrt{\textrm{dcov}_{ii} \textrm{dcov}_{kk}}} \right) \right] , \quad k = 1, 2, \ldots , m. \end{aligned}$$

In this definition, the fraction $\textrm{dcov}_{ik}/\sqrt{\textrm{dcov}_{ii} \textrm{dcov}_{kk}}$ is the sample distance correlation coefficient between criteria *i* and *k*. This coefficient measures the degree of dependency, which can capture both linear and nonlinear relationships between the indicators. The integration of the distance correlation coefficient into the CRITIC method eliminates the need to address the positivity or negativity of the correlation coefficients, thereby resolving issues inherent to the original CRITIC method. Previously, the CRITIC method tended to assign disproportionately greater weights to criteria exhibiting negative correlations, leading to undue emphasis and potential data redundancy.

With the introduction of the distance correlation coefficient, the method can more effectively capture both the correlation and independence between criteria. This enhancement allows for a more nuanced weighting approach, reducing weights for criteria with high data redundancy. Consequently, this adjustment ensures a fairer distribution across all criteria and minimizes data redundancy, thereby improving the overall robustness and fairness of the CRITIC method.

## Simulated numerical experiments

### Efficacy

We conducted simulations to assess the efficacy of the CRITID-Dcor method relative to the original CRITIC method under both nonlinear and linear conditions. The simulations involved three random variables, *X*, *Y*, and *Z*. The relationship between *Y* and *X* varied across different scenarios, while *Z* and *X* were independent and identically distributed (i.i.d).

First, we provide a rationale for the allocation of optimal prior weights in our simulated data. In the scenarios we simulated, *X* and *Y* exhibit a highly nonlinear relationship, whereas *Z* is independent of both *X* and *Y*. According to the principles of the CRITIC method, the initial weights for the indicators *X*, *Y*, and *Z* should ideally be in the ratio 1:1:2, reflecting weights of 0.25, 0.25, and 0.5 respectively. This weighting is derived from the formula $\sum _{i=1}^m \left( 1-r_{ik}\right)$ where $r_{ik}$ approaches 1 for nearly perfect correlation.

Moreover, it is essential to consider not only the correlation but also the variability of each indicator. Assuming the standard deviations of *X*, *Y*, and *Z* are *sd*(*X*), *sd*(*Y*), and *sd*(*Z*) respectively, the weights calculated by formula 1.1 adjust for this variability. Thus, the weights of criteria *X*, *Y*, and *Z* are determined as follows:

$$\begin{aligned} & \omega _X = \frac{0.25 \cdot sd(X)}{0.25 \cdot sd(X) + 0.25 \cdot sd(Y) + 0.5 \cdot sd(Z)},\\ & \omega _Y = \frac{0.25 \cdot sd(Y)}{0.25 \cdot sd(X) + 0.25 \cdot sd(Y) + 0.5 \cdot sd(Z)},\\ & \omega _Z = \frac{0.5 \cdot sd(Z)}{0.25 \cdot sd(X) + 0.25 \cdot sd(Y) + 0.5 \cdot sd(Z)}. \end{aligned}$$

We present several examples to illustrate how standard deviations influence the determination of optimal prior weights:

- **Equal Standard Deviations:** If $sd(X) \approx sd(Y) \approx sd(Z)$, then the weights should align such that $\omega _X \approx \omega _Y \ll \omega _Z$. This reflects the higher relative importance assigned to *Z*, in accordance with its greater initial weighting ratio (0.5 compared to 0.25 for *X* and *Y*).
- **Greater Variation in** *X* **and** *Y*: If $sd(X) \approx sd(Y) \gg sd(Z)$, then the weights should be $\omega _X \approx \omega _Y$ and combined, $\omega _X + \omega _Y \gg \omega _Z$. This scenario highlights the dominance of *X* and *Y* due to their greater variability compared to *Z*.
- **Greater Variation in** *X* **and** *Z*: If $sd(X) \approx sd(Z) \gg sd(Y)$, the optimal prior weights should be such that $\omega _X \ll \omega _Z$, $\omega _Z \gg \omega _Y$, and $\omega _X \gg \omega _Y$. In this case, both *X* and *Z* are significantly more variable than *Y*, leading to greater weights for *X* and *Z* compared to *Y*.

The remaining cases can be deduced based on the optimal prior weights described above. It is important to note that the weights provided here are qualitative and not quantitative. A weight is considered reasonable if it closely approximates the specified optimal prior weights and adheres to the described relationships among the weights.

All examples were replicated 500 times to ensure the robustness and reliability of the results. This repetition helps to validate the consistency of the weights under various scenarios and standard deviations. All data have been min-max normalized to ensure comparability across different scales.

#### Example 1.1

 Consider a scenario where $Y = \ln |X| + \varepsilon$, with $X, Z \sim N(0,1)$ and $\varepsilon \sim N(0,0.001)$.

**Figure 2:**
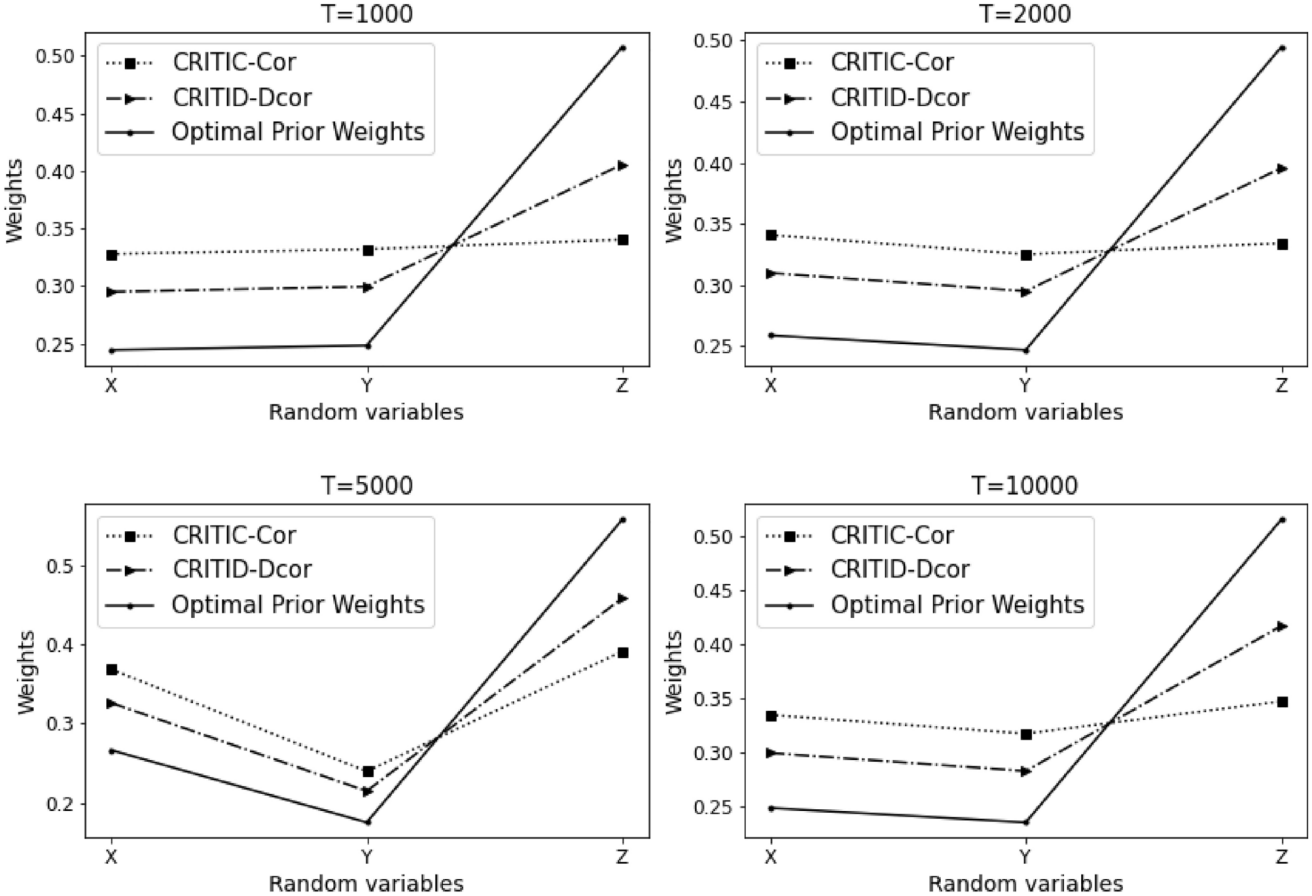
Detailed comparison and analysis results for Example 1.1.

The results of Example 1.1 are summarized in Table 1 and Fig. 2 presented below. Here, $T$ denotes the sample size, *CRITIC-Cor* represents the original CRITIC method, and *CRITID-Dcor* denotes the improved method. The results for Example 1.1 are presented in Table 1.

**Table 1:** Comparative analysis of weights calculated by CRITIC-Cor and CRITID-Dcor methods against optimal prior weights across different sample sizes in Example 1.1.

T	Mean standard deviation			Optimal prior weight			CRITIC-Cor weight			CRITID-Dcor weight		
	X	Y	Z	X	Y	Z	X	Y	Z	X	Y	Z
1000	0.150	0.153	0.156	0.245	0.248	0.508	0.328	0.332	0.340	0.295	0.300	0.406
2000	0.143	0.137	0.137	0.259	0.247	0.495	0.341	0.325	0.334	0.310	0.295	0.396
5000	0.137	0.090	0.143	0.267	0.175	0.558	0.369	0.240	0.391	0.326	0.215	0.459
10000	0.121	0.114	0.126	0.249	0.235	0.516	0.335	0.318	0.348	0.300	0.283	0.418

Weights depicted in Fig. 2 are represented as solid lines, indicating the theoretical optimal prior weights. A reasonable weight should align closely with these solid lines. When observing the average standard deviation of the three criteria at a sample size of 1000, the standard deviations are almost identical. Based on the proposed optimal prior weights, the weights of indicators *X* and *Y* should be approximately equal, whereas the weight of indicator *Z* should be significantly higher than that of the first two.

It is evident that the weights assigned by the CRITIC-Cor method are not justifiable, as the weight of indicator *X* exceeds that of indicator *Z*, contradicting the expected ratios. Conversely, the weights determined by the CRITID-Dcor method are more aligned with expectations, where the weight of indicator *Z* is substantially greater than those of *X* and *Y*, and the weights of *X* and *Y* are approximately equal.

Moreover, the proximity of the weights of *X*, *Y*, and *Z* to the optimal prior weights in the figure improves as the sample size increases. The weights assigned by the CRITID-Dcor method consistently approximate the optimal prior weights, irrespective of the sample size. This is in stark contrast to the CRITIC-Cor method, which consistently yields unreasonable weights. For example, at a sample size of 10,000, where the average standard deviations are similar, optimal prior weights or “reasonable” weights would be close to 0.25, 0.25, and 0.5. The weights provided by the CRITID-Dcor method are closely aligned with these figures, whereas the weights from CRITIC-Cor are closer to 0.33, 0.33, 0.33, deviating significantly from the expected distribution.

We also calculated the Mean Squared Error (MSE) of the weights derived using the CRITIC-Cor method and the CRITID-Dcor method, both compared to optimal prior weights. The results from Example 1.1 reveal that the MSE for CRITIC-Cor is 1.380%, while the MSE for CRITID-Dcor is significantly lower, at only 0.499%.

#### Example 1.2

 Let $Y=\ln |X|+\varepsilon$, where $X, Z$ is assumed to follow an Exponential distribution with a rate parameter of 1 ($X, Z \sim \text {E}(1)$), $\varepsilon \sim N(0,0.001)$.

**Figure 3:**
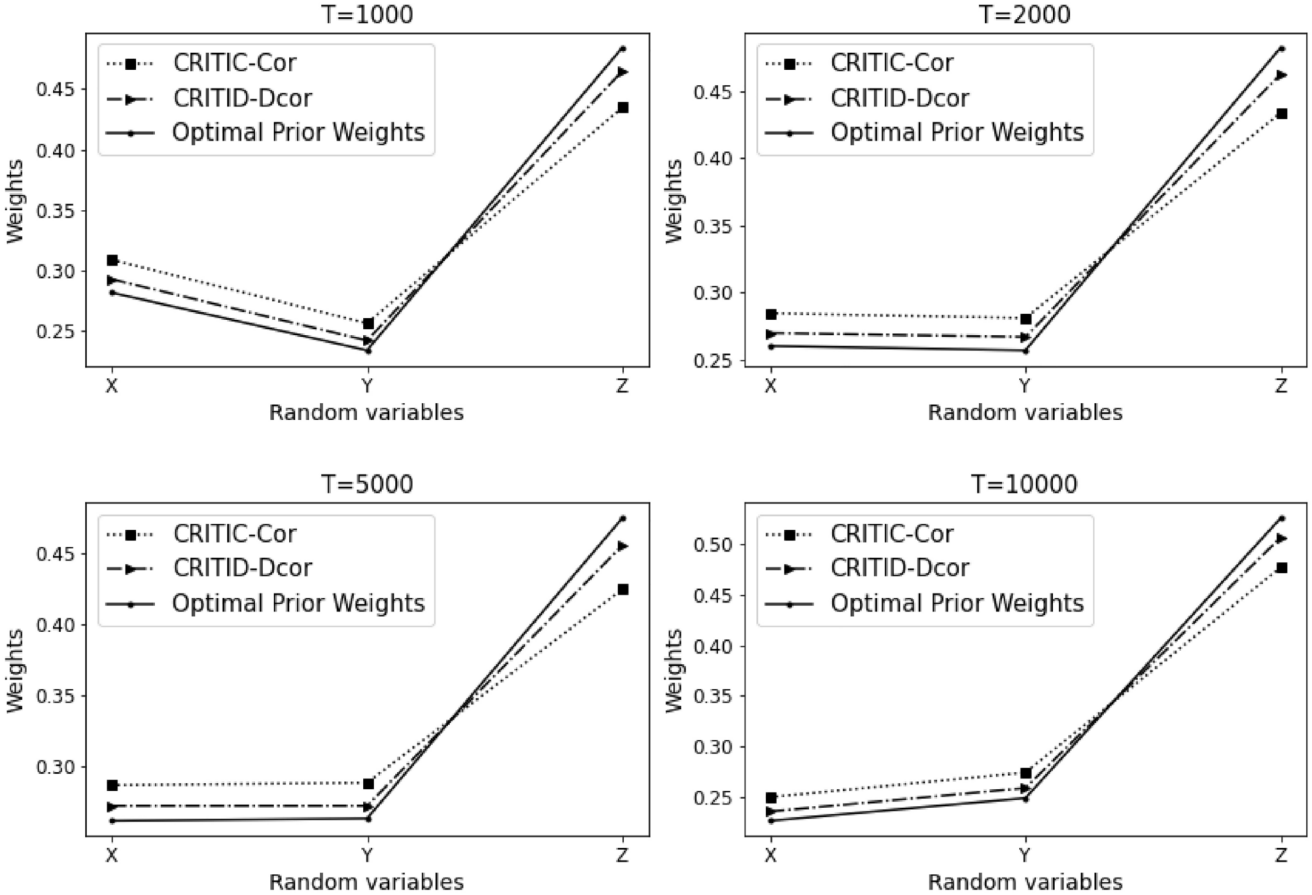
Detailed comparison and analysis results for Example 1.2.

The results of Example 1.2 are summarized in Table 2 and Fig. 3 presented below. In this example, the relationship between indicators *X* and *Y* exhibits a relatively high degree of linearity. This is attributed to the fact that values generated from an exponential distribution are always positive, and the nonlinear transformation applied, $\ln |X|$, is a monotonically increasing function over this range.

**Table 2:** Comparative analysis of weights calculated by CRITIC-Cor and CRITID-Dcor methods against optimal prior weights across different sample sizes in Example 1.2.

T	Mean standard deviation			Optimal prior weight			CRITIC-Cor weight			CRITID-Dcor weight		
	X	Y	Z	X	Y	Z	X	Y	Z	X	Y	Z
1000	0.164	0.136	0.141	0.282	0.234	0.484	0.309	0.256	0.435	0.293	0.242	0.465
2000	0.125	0.123	0.116	0.261	0.257	0.482	0.285	0.281	0.434	0.270	0.267	0.463
5000	0.113	0.113	0.102	0.262	0.263	0.475	0.287	0.289	0.425	0.272	0.272	0.456
10000	0.100	0.110	0.117	0.226	0.248	0.526	0.249	0.274	0.477	0.235	0.258	0.507

Observations indicate that with a sample size of 1000, the weights assigned by both the CRITIC-Cor and the CRITID-Dcor methods appear reasonable. As the sample size increases, the weights provided by both methods continue to be justifiable, and the average standard deviations of the three indicators converge closely together. In this scenario, both methods tend to yield optimal prior weights of 0.25, 0.25, and 0.5. Consequently, in this specific example, the weights calculated by both the CRITIC-Cor method and the CRITID-Dcor method are considered reasonable.

However, it is noteworthy that the weights calculated by the CRITID-Dcor method consistently approximate the optimal prior weights or ’reasonable’ weights more closely than those calculated by the CRITIC-Cor method. This suggests a superior alignment of the CRITID-Dcor method with the expected outcomes under varying sample sizes. In Example 1.2, the MSE for the CRITIC-Cor method is 0.121%, while the MSE for the CRITID-Dcor method is significantly lower at 0.019%.

#### Example 1.3

 Let $Y=\ln |X|+\varepsilon$, where *X*, *Z* is assumed to follow an uniform distribution over the interval from -10 to 10 ($X, Z \sim U(-10,10)$), $\varepsilon \sim N(0,0.001)$.

**Figure 4:**
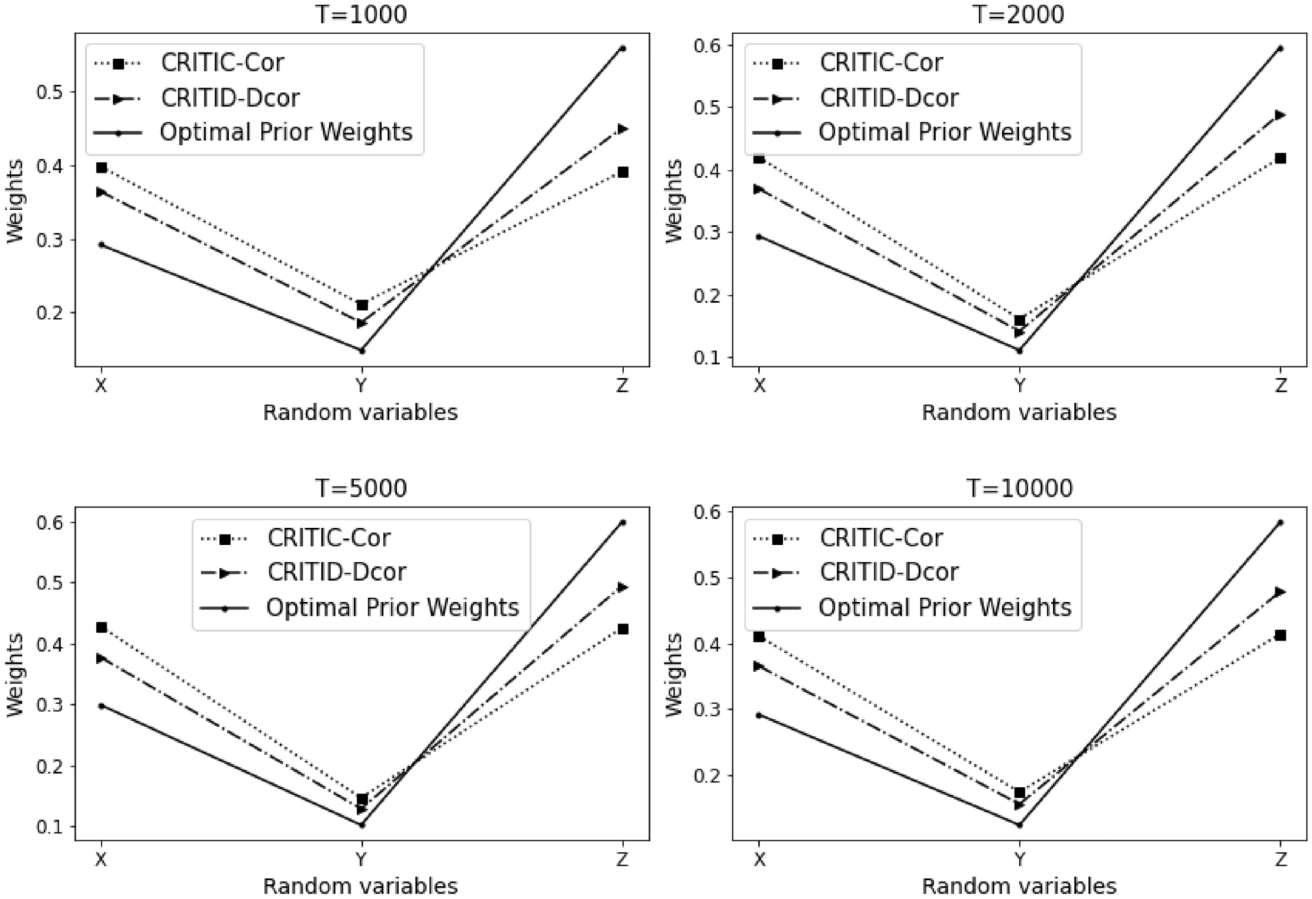
Detailed comparison and analysis results for Example 1.3.

The results of Example 1.3 are summarized in Table 3 and Fig. 4 presented below.

**Table 3:** Comparative analysis of weights calculated by CRITIC-Cor and CRITID-Dcor methods against optimal prior weights across different sample sizes in Example 1.3.

T	Mean standard deviation			Optimal prior weight			CRITIC-Cor weight			CRITID-Dcor weight		
	X	Y	Z	X	Y	Z	X	Y	Z	X	Y	Z
1000	0.295	0.150	0.282	0.292	0.149	0.559	0.398	0.211	0.391	0.364	0.186	0.450
2000	0.287	0.109	0.291	0.294	0.111	0.595	0.420	0.161	0.419	0.369	0.142	0.489
5000	0.289	0.099	0.290	0.299	0.102	0.599	0.428	0.147	0.425	0.377	0.129	0.494
10000	0.289	0.123	0.289	0.292	0.124	0.584	0.412	0.174	0.414	0.366	0.156	0.479

Similar to Example 1.1, in this scenario, indicators *X* and *Y* exhibit a high degree of nonlinearity. Considering a sample size of 1000, the standard deviations of indicators *X* and *Z* are similar. Based on the optimal prior weights previously established, the weight of indicator *Z* should be significantly greater than that of indicator *X*. The CRITIC-Cor method assigns weights to *X* and *Z* that are relatively close, which is inconsistent with the expected distribution based on their standard deviations and the non-linear relationship between *X* and *Y*. In contrast, the CRITID-Dcor method assigns a much greater weight to indicator *Z* compared to *X*, aligning more closely with the theoretical expectations.

This pattern holds as the sample size increases. The CRITIC-Cor method continues to provide weights that do not reflect the nonlinearity and independence between *X* and *Y*, consistently proving to be unreasonable. Conversely, the CRITID-Dcor method remains reasonable across different sample sizes. Notably, at a sample size of 10,000, CRITIC-Cor assigns almost equal weights to *X* and *Z*, with both nearing 0.5. This occurs because the Pearson correlation coefficient used in CRITIC-Cor cannot adequately capture the nonlinear relationship between *X* and *Y*, leading to a misjudgment of their independence. Additionally, since the average standard deviations of *X* and *Z* are very close, and significantly larger than that of *Y*, this results in the disproportionately high weights for *X* and *Z* observed under the CRITIC-Cor method. In Example 1.3, the MSE for CRITIC-Cor is noted at 1.563%, compared to a significantly lower MSE of 0.599% for CRITID-Dcor.

#### Example 2.1

 Let $Y=7X^6-5X^4+3X^2-X+\varepsilon$, where $X, Z \sim N(0,1)$, and $\varepsilon \sim N(0,0.001)$.

**Figure 5:**
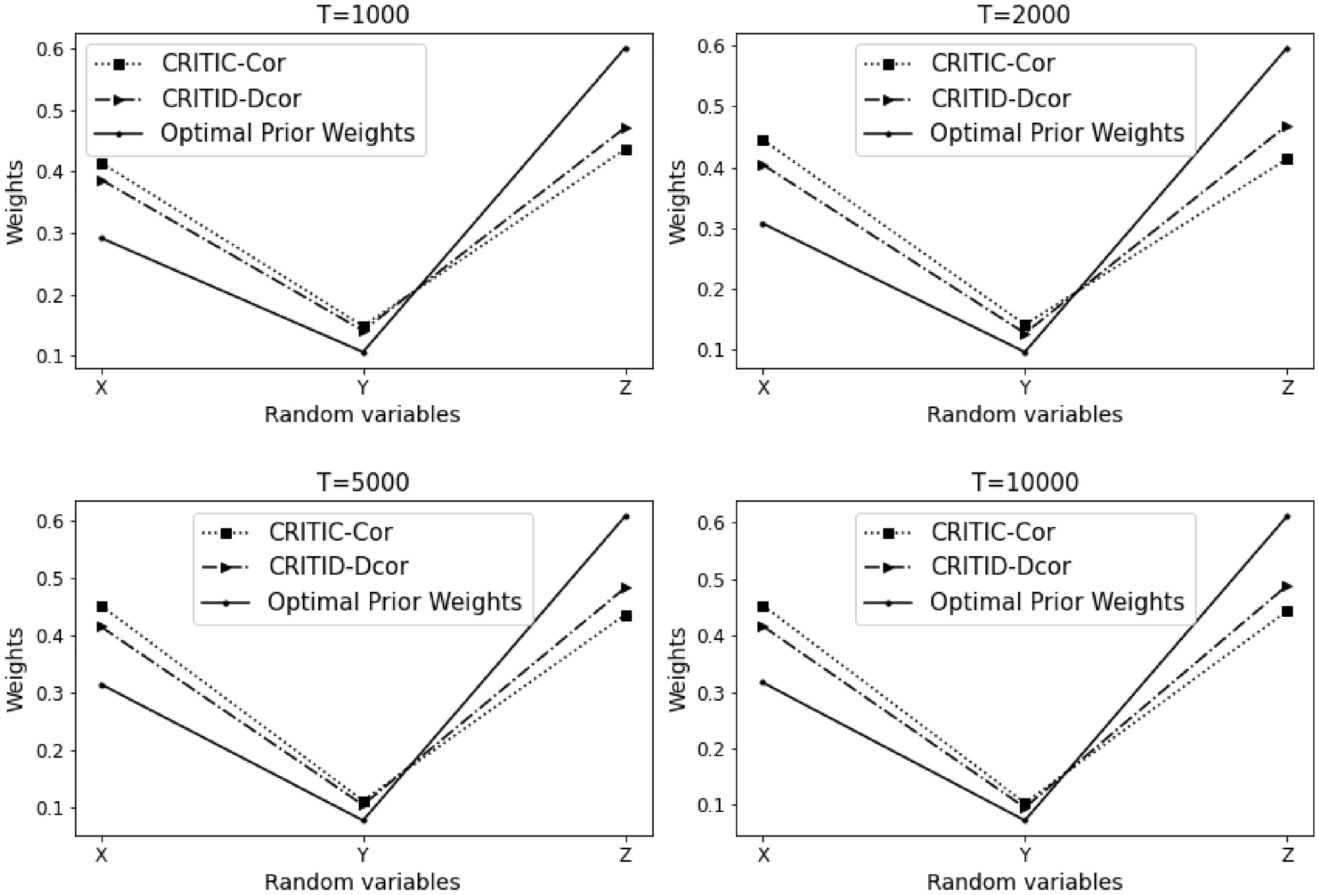
Detailed comparison and analysis results for Example 2.1.

The results of Example 2.1 are summarized in Table 4 and Fig. 5 presented below.

**Table 4:** Comparative analysis of weights calculated by CRITIC-Cor and CRITID-Dcor methods against optimal prior weights across different sample sizes in Example 2.1.

T	Mean standard deviation			Optimal prior weight			CRITIC-Cor weight			CRITID-Dcor weight		
	X	Y	Z	X	Y	Z	X	Y	Z	X	Y	Z
1000	0.148	0.054	0.152	0.292	0.107	0.601	0.415	0.149	0.436	0.387	0.141	0.472
2000	0.147	0.046	0.142	0.308	0.096	0.596	0.445	0.142	0.414	0.404	0.127	0.469
5000	0.138	0.034	0.133	0.315	0.077	0.608	0.452	0.112	0.436	0.415	0.104	0.483
10000	0.134	0.031	0.129	0.317	0.073	0.611	0.453	0.104	0.443	0.416	0.096	0.488

In this example, the average standard deviations of *X* and *Z* are approximately equal, while the average standard deviation of *Y* is significantly smaller than those of *X* and *Z*. Based on the optimal prior weights we previously established, the weight of *Z* should be substantially greater than that of *X*, and both *X* and *Z* should receive much higher weights compared to *Y*.

The results indicate that the CRITIC-Cor method does not align with these theoretical weight distributions, failing to adequately reflect the differences in variability between the indicators. In contrast, the CRITID-Dcor method assigns weights that more accurately match the expected values, recognizing the disparity in standard deviations and assigning weights accordingly.

Thus, while the CRITIC-Cor method falls short of meeting the stipulated optimal prior weights, the CRITID-Dcor method successfully adheres to them, proving its effectiveness in incorporating the actual dynamics observed among the indicators. For Example 2.1, the MSE values are 1.629% for CRITIC-Cor and 0.879% for CRITID-Dcor.

#### Example 2.2

 Let $Y=7X^6-5X^4+3X^2-X+\varepsilon$, where $X, Z \sim \text {E}(1)$, and $\varepsilon \sim N(0,0.001)$.

**Figure 6:**
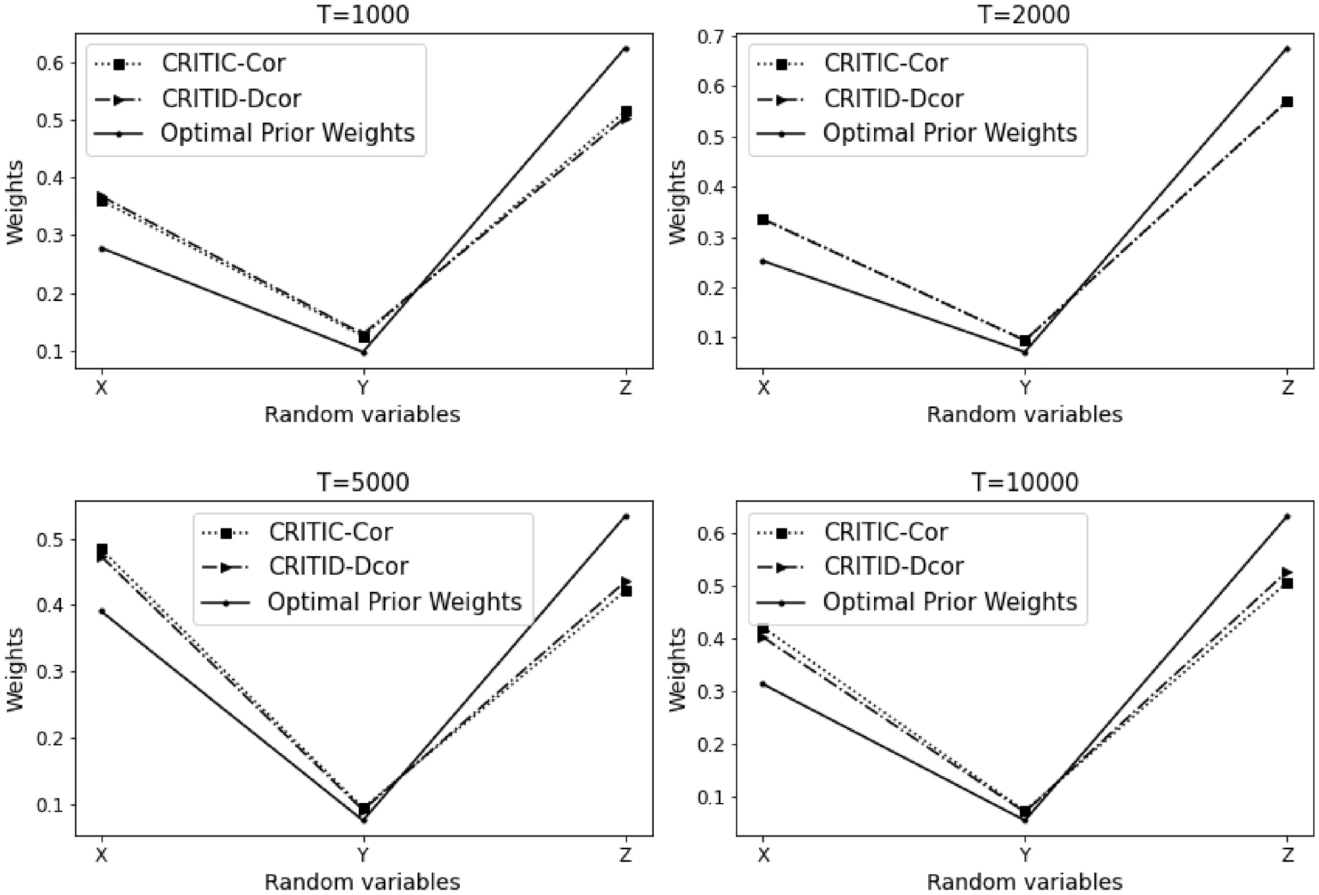
Detailed comparison and analysis results for Example 2.2.

The results of Example 2.2 are summarized in Table 5 and Fig. 6 presented below. In this example, the weights calculated by both the CRITIC-Cor method and the CRITID-Dcor method are very similar and are generally deemed reasonable. However, there exists an apparent anomaly where the relationship expected by the optimal prior weights is not strictly adhered to. This situation prompts the question of why these weights are still considered reasonable despite the violation of the expected relationship.

**Table 5:** Comparative analysis of weights calculated by CRITIC-Cor and CRITID-Dcor methods against optimal prior weights across different sample sizes in Example 2.2.

T	Mean standard deviation			Optimal prior weight			CRITIC-Cor weight			CRITID-Dcor weight		
	X	Y	Z	X	Y	Z	X	Y	Z	X	Y	Z
1000	0.114	0.040	0.128	0.278	0.097	0.625	0.360	0.125	0.515	0.368	0.128	0.504
2000	0.120	0.034	0.161	0.253	0.071	0.677	0.337	0.094	0.569	0.336	0.094	0.570
5000	0.128	0.025	0.087	0.391	0.076	0.534	0.485	0.094	0.421	0.474	0.091	0.435
10000	0.107	0.019	0.108	0.314	0.055	0.632	0.421	0.073	0.506	0.403	0.070	0.527

The key to understanding this lies in the significantly smaller standard deviation of indicator *Y*. Because *Y*’s standard deviation is minimal, its influence on the overall weighting can be considered almost negligible. Consequently, the weights of criteria *X* and *Z* are primarily determined by their own standard deviations, largely independent of their correlation with *Y*. This leads to a scenario where the weights for *X* and *Z*, as calculated by both methods, are nearly identical and reasonable. The minimal impact of *Y* on the weighting system allows both methods to produce similar outcomes, which align closely enough with the theoretical expectations to be considered reasonable, despite the slight deviation from the ideal relationship among the weights. In Example 2.2, MSE outcomes demonstrate that CRITIC-Cor stands at 0.736%, closely followed by CRITID-Dcor with 0.653%.

#### Example 2.3

 Let $Y=7X^6-5X^4+3X^2-X+\varepsilon$, where $X, Z \sim U(-10,10)$, and $\varepsilon \sim N(0,0.001)$.

**Figure 7:**
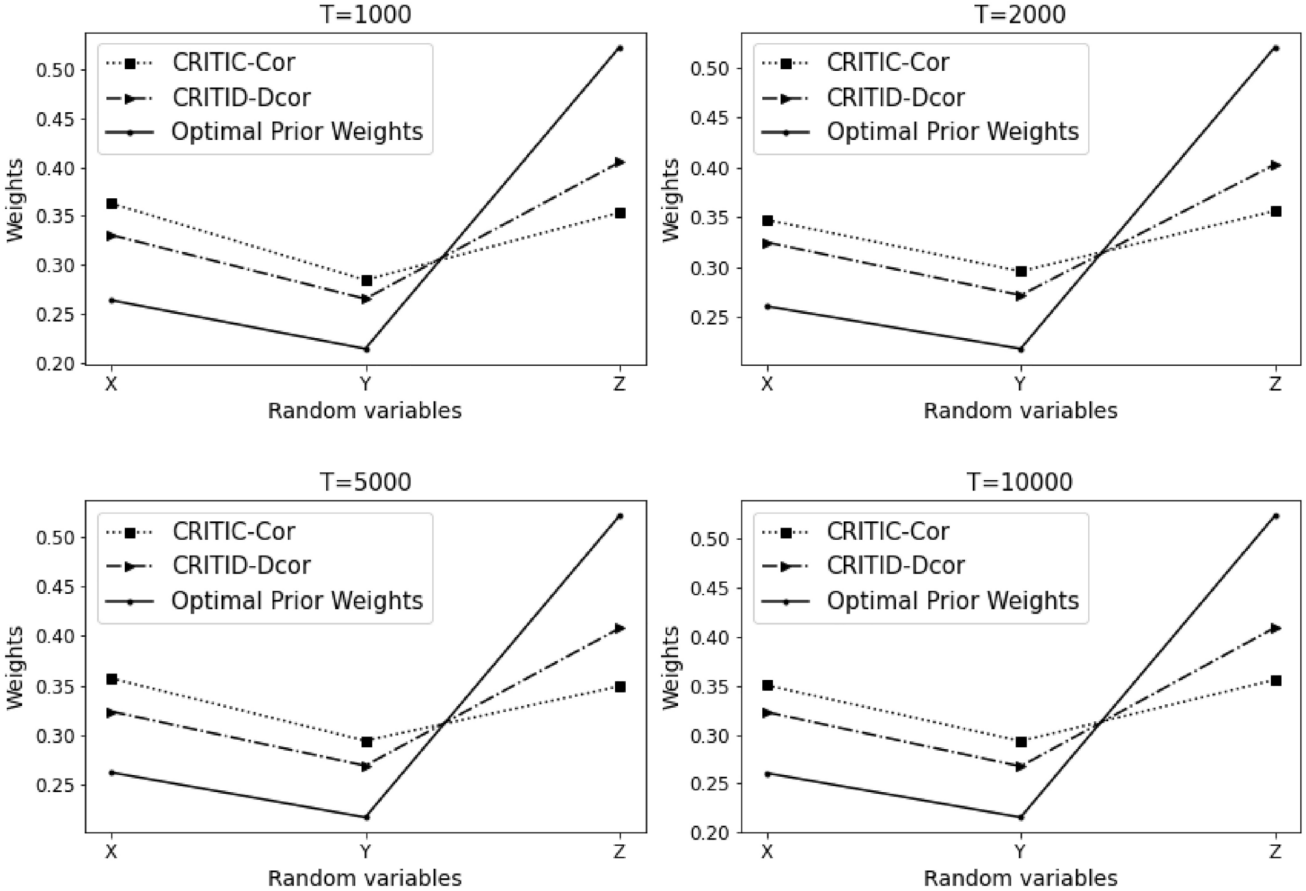
Detailed comparison and analysis results for Example 2.3.

The results of Example 2.3 are summarized in Table 6 and Fig. 7 presented below.

**Table 6:** Comparative analysis of weights calculated by CRITIC-Cor and CRITID-Dcor methods against optimal prior weights across different sample sizes in Example 2.3.

T	Mean standard deviation			Optimal prior weight			CRITIC-Cor weight			CRITID-Dcor weight		
	X	Y	Z	X	Y	Z	X	Y	Z	X	Y	Z
1000	0.288	0.234	0.286	0.264	0.214	0.523	0.363	0.284	0.353	0.330	0.265	0.405
2000	0.290	0.243	0.288	0.261	0.219	0.520	0.348	0.296	0.356	0.325	0.272	0.403
5000	0.288	0.238	0.287	0.262	0.216	0.522	0.357	0.294	0.349	0.324	0.269	0.408
10000	0.286	0.237	0.288	0.261	0.215	0.524	0.351	0.293	0.356	0.323	0.268	0.410

This example shares similarities with Example 1.3, where the performance of the CRITID-Dcor method is consistently reasonable, whereas the CRITIC-Cor method repeatedly fails to meet the expected standards. Specifically, the CRITIC-Cor method calculates a weight for indicator *X* that is greater than or roughly equal to the weight of *Z*, which directly contradicts the predefined optimal prior weights.

The persistent issue with CRITIC-Cor is that it does not accurately account for the relative importance that should be ascribed based on the variability and correlation characteristics of the criteria. In stark contrast, the CRITID-Dcor method correctly prioritizes the weights according to the established criteria, adhering to the reasonable weight guidelines, thereby demonstrating its reliability and consistency in scenarios where CRITIC-Cor struggles. Example 2.3 reveals an MSE of 1.425% for CRITIC-Cor, while CRITID-Dcor achieves a notably lower MSE of 0.674%.

#### Example 3.1

 Let $Y=2\cdot e^{-\frac{X^2}{2}}+\varepsilon$, where $X, Z \sim N(0,1)$, and $\varepsilon \sim N(0,0.001)$.

**Figure 8:**
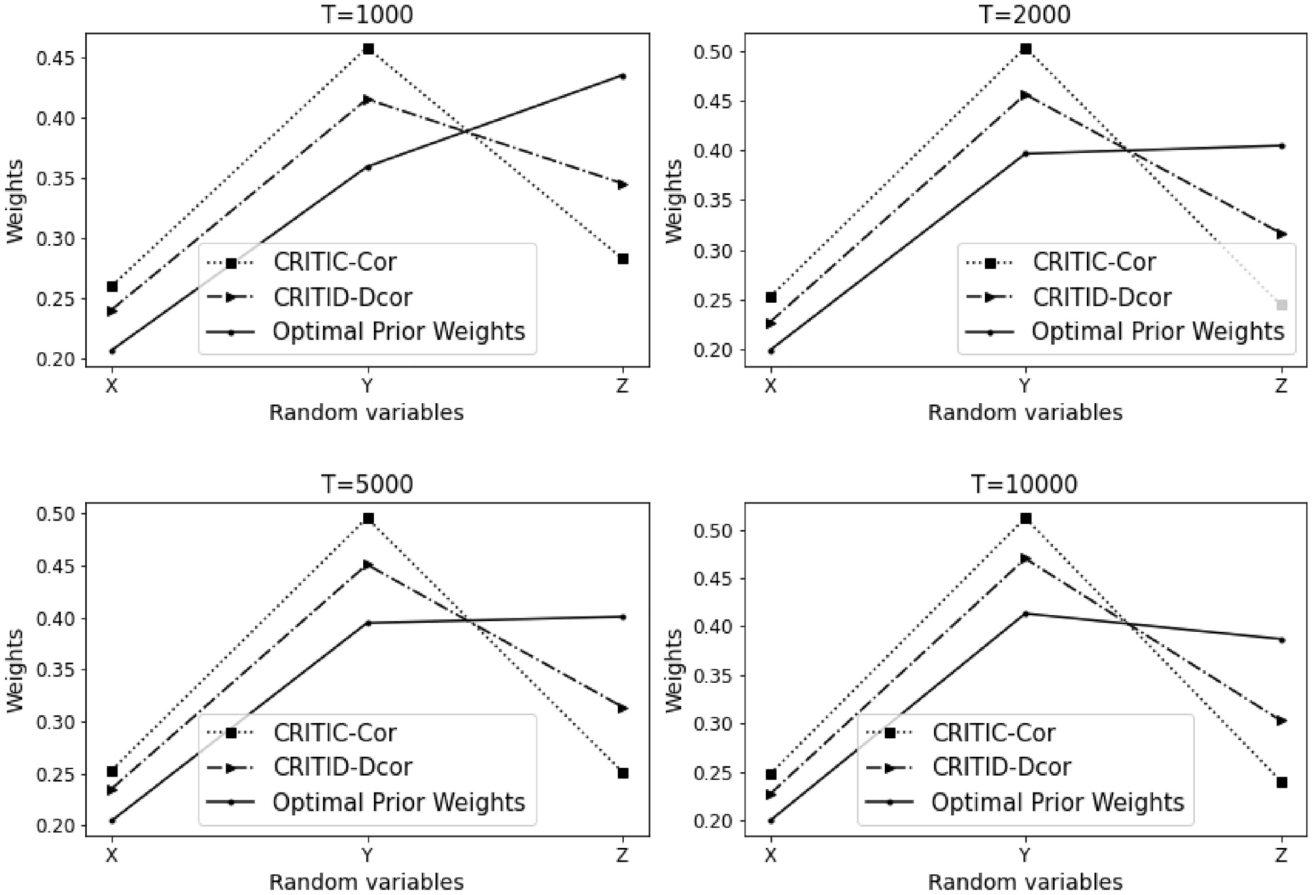
Detailed comparison and analysis results for Example 3.1.

The results of Example 3.1 are summarized in Table 7 and Fig. 8 presented below.

**Table 7:** Comparative analysis of weights calculated by CRITIC-Cor and CRITID-Dcor methods against optimal prior weights across different sample sizes in Example 3.1.

T	Mean standard deviation			Optimal prior weight			CRITIC-Cor weight			CRITID-Dcor weight		
	X	Y	Z	X	Y	Z	X	Y	Z	X	Y	Z
1000	0.161	0.280	0.170	0.206	0.359	0.435	0.260	0.458	0.283	0.240	0.415	0.345
2000	0.140	0.279	0.142	0.199	0.397	0.405	0.252	0.504	0.244	0.227	0.457	0.317
5000	0.145	0.280	0.142	0.204	0.395	0.401	0.253	0.496	0.251	0.235	0.451	0.314
10000	0.134	0.278	0.130	0.200	0.413	0.387	0.248	0.512	0.241	0.227	0.470	0.303

In this scenario, with a sample size of 1000, the mean standard deviations of *X* and *Z* are observed to be approximately equal. According to the established guidelines for optimal prior weights, the weight of *Z* should significantly exceed that of *X*. However, while the CRITID-Dcor method appropriately reflects this expectation, providing a reasonable weighting consistent with the guidelines, the CRITIC-Cor method fails to do so, marking it as unreasonable.

As the sample size increases, the flaws in the CRITIC-Cor method become increasingly evident, with its weight assignments diverging further from what is considered reasonable. Conversely, the CRITID-Dcor method maintains its consistency and reasonableness across larger sample sizes. This consistency highlights the robustness of the CRITID-Dcor method in accurately reflecting the relationships and variabilities among the indicators, whereas the CRITIC-Cor method’s limitations become more pronounced, undermining its effectiveness in scenarios requiring accurate weighting based on variability and correlation characteristics. The MSE for CRITIC-Cor is 1.203%, and the MSE for CRITID-Dcor is 0.393% in Example 3.1.

#### Example 3.2

 Let $Y=2\cdot e^{-\frac{X^2}{2}}+\varepsilon$, where $X, Z \sim \text {E}(1)$, and $\varepsilon \sim N(0,0.001)$.

**Figure 9:**
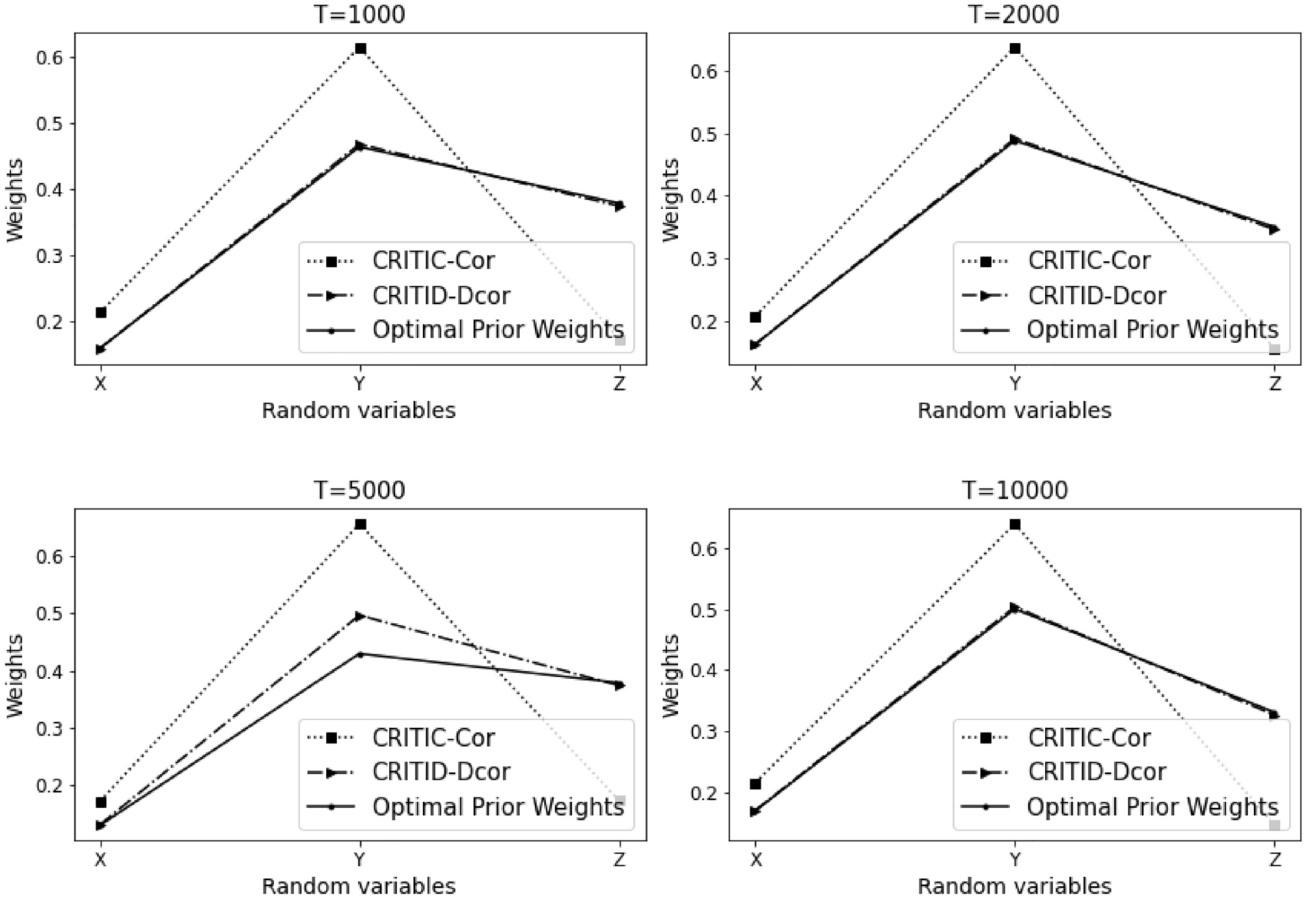
Detailed comparison and analysis results for Example 3.2.

The results of Example 3.2 are summarized in Table 8 and Fig. 9 presented below.

**Table 8:** Comparative analysis of weights calculated by CRITIC-Cor and CRITID-Dcor methods against optimal prior weights across different sample sizes in Example 3.2.

T	Mean standard deviation			Optimal prior weight			CRITIC-Cor weight			CRITID-Dcor weight		
	X	Y	Z	X	Y	Z	X	Y	Z	X	Y	Z
1000	0.119	0.349	0.142	0.158	0.463	0.378	0.213	0.615	0.173	0.159	0.468	0.374
2000	0.112	0.338	0.121	0.162	0.488	0.350	0.206	0.639	0.155	0.162	0.492	0.346
5000	0.089	0.338	0.130	0.130	0.429	0.378	0.172	0.655	0.173	0.131	0.496	0.373
10000	0.115	0.342	0.113	0.169	0.500	0.331	0.215	0.640	0.145	0.170	0.504	0.327

In this example, the performance of the CRITIC-Cor method consistently falls short of expectations, rendering it unreasonable throughout the evaluations. In stark contrast, the weight obtained by the CRITID-Dcor method aligns closely with the theoretically defined optimal prior weight, demonstrating its effectiveness almost invariably. This consistent accuracy in weight assignment by the CRITID-Dcor method underscores its robustness and reliability in adhering to the established criteria for reasonable weighting, in contrast to the persistent inadequacies observed with the CRITIC-Cor method. In Example 3.2, the MSE for CRITIC-Cor is significantly higher at 2.350%, in contrast to a much lower MSE of 0.038% for CRITID-Dcor.

#### Example 3.3

 Let $Y=2\cdot e^{-\frac{X^2}{2}}+\varepsilon$, where $X, Z$ is modeled as following a Poisson distribution with a parameter of 1 ($X, Z \sim \text {P}(1)$), and $\varepsilon \sim N(0,0.001)$.

**Figure 10:**
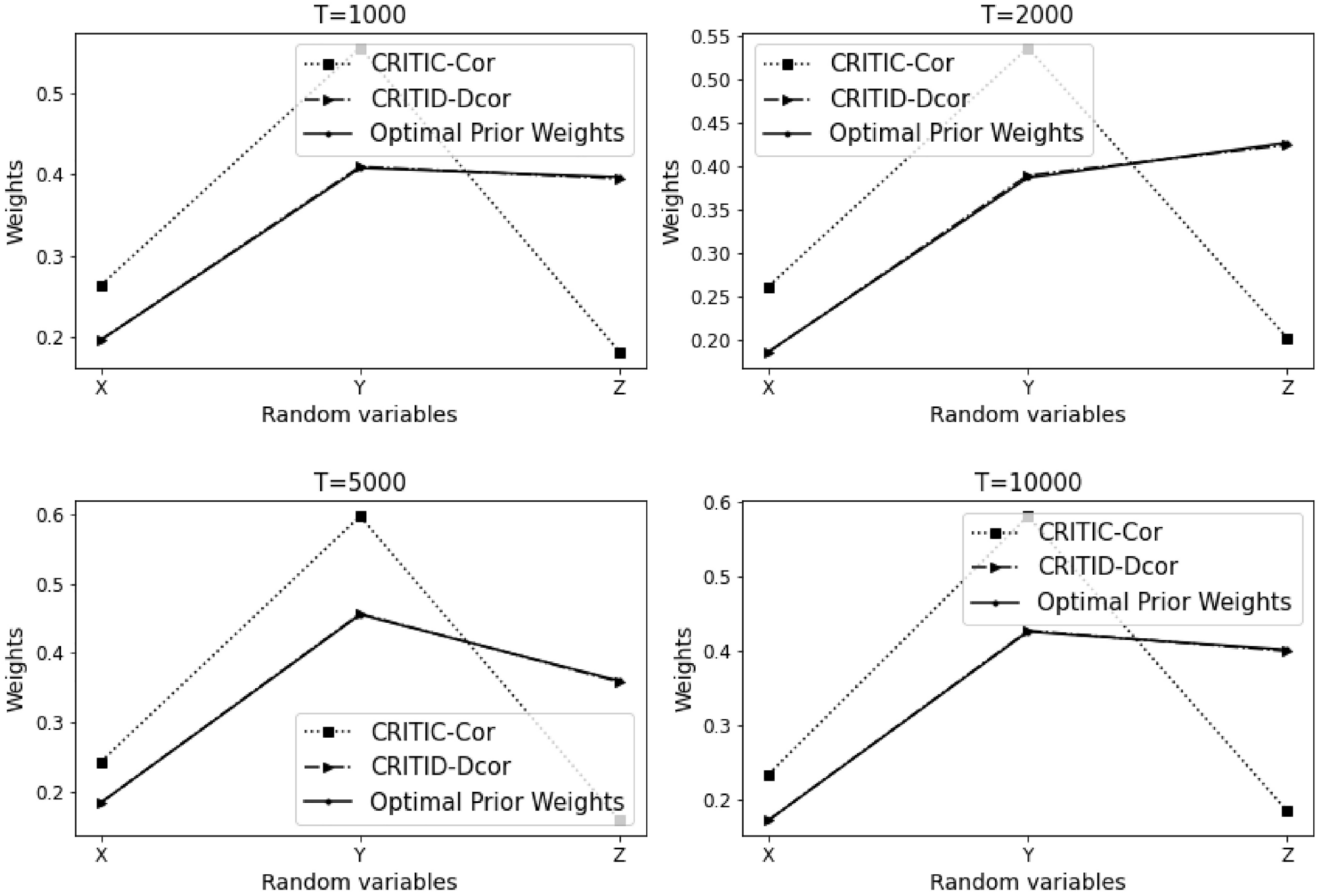
Detailed comparison and analysis results for Example 3.3.

The results of Example 3.3 are summarized in Table 9 and Fig. 10 presented below.

**Table 9:** Comparative analysis of weights calculated by CRITIC-Cor and CRITID-Dcor methods against optimal prior weights across different sample sizes in Example 3.3.

T	Mean standard deviation			Optimal prior weight			CRITIC-Cor weight			CRITID-Dcor weight		
	X	Y	Z	X	Y	Z	X	Y	Z	X	Y	Z
1000	0.172	0.359	0.175	0.196	0.408	0.397	0.263	0.556	0.181	0.196	0.409	0.395
2000	0.175	0.363	0.200	0.186	0.387	0.427	0.262	0.536	0.202	0.186	0.389	0.424
5000	0.145	0.358	0.142	0.185	0.455	0.360	0.244	0.597	0.160	0.185	0.456	0.359
10000	0.144	0.355	0.168	0.173	0.426	0.402	0.234	0.581	0.185	0.173	0.427	0.400

This example bears close resemblance to Example 3.1, where the performance of the CRITID-Dcor method consistently approaches the theoretically established reasonable weight. This consistency underscores the method’s robustness and accuracy in weighting indicators based on their inherent characteristics and interrelationships.

In contrast, the CRITIC-Cor method consistently fails to meet these criteria, proving to be unreasonable across various scenarios. The persistent failure of the CRITIC-Cor method to align with optimal prior weights highlights fundamental shortcomings in its approach to handling the complexities of indicator relationships, especially when dealing with non-linear correlations and variable standard deviations. Example 3.3 demonstrates a significant disparity in MSE results: CRITIC-Cor records an MSE of 2.415%, whereas CRITID-Dcor achieves an MSE of nearly 0.000%, indicating near-perfect performance.

#### Example 3.4

 Let $Y=2\cdot e^{-\frac{X^2}{2}}+\varepsilon$, where $X, Z \sim U(-10,10)$, and $\varepsilon \sim N(0,0.001)$.

**Figure 11:**
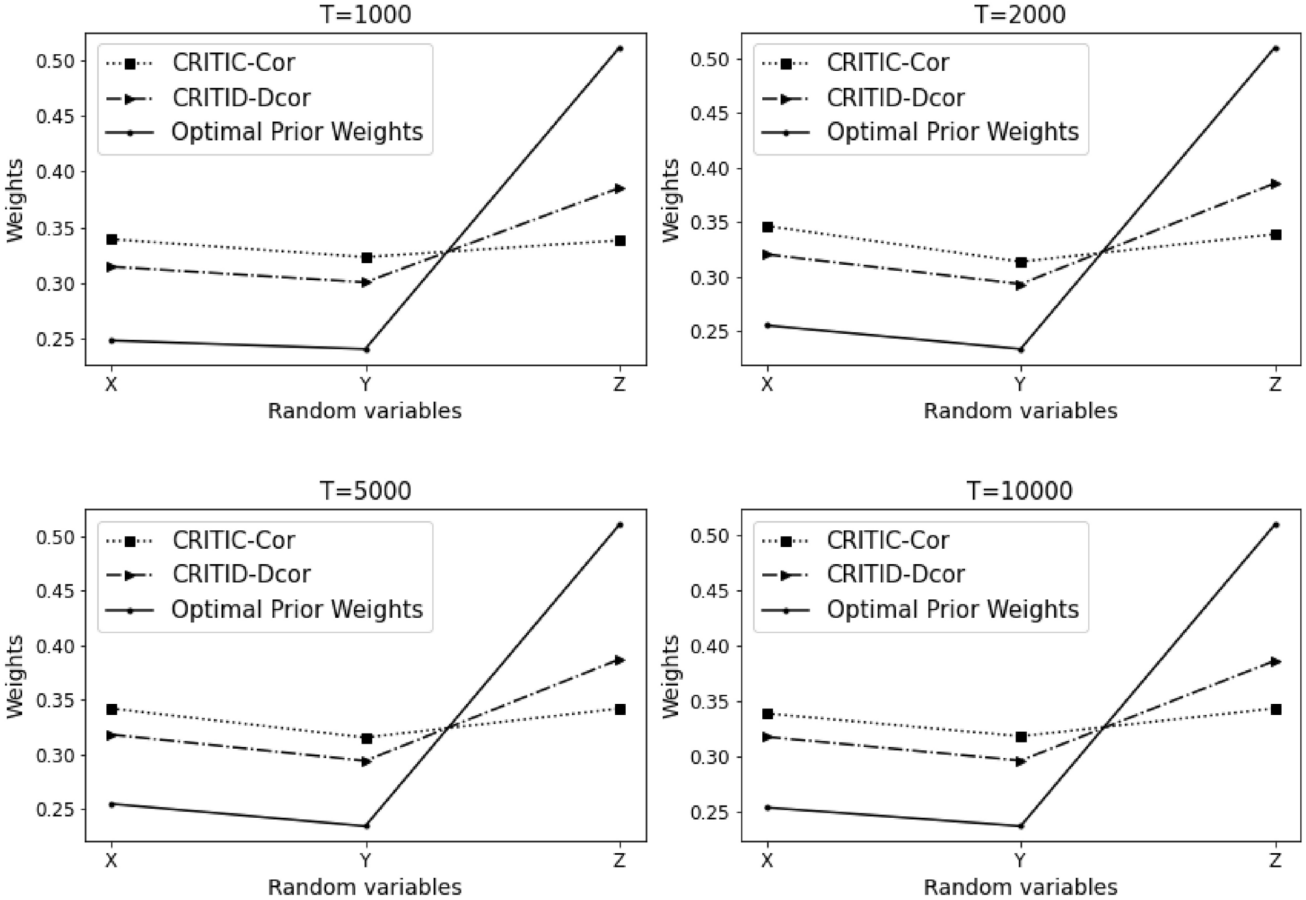
Detailed comparison and analysis results for Example 3.4.

The results of Example 3.4 are summarized in Table 10 and Fig. 11 presented below.

**Table 10:** Comparative analysis of weights calculated by CRITIC-Cor and CRITID-Dcor methods against optimal prior weights across different sample sizes in Example 3.4.

T	Mean standard deviation			Optimal prior weight			CRITIC-Cor weight			CRITID-Dcor weight		
	X	Y	Z	X	Y	Z	X	Y	Z	X	Y	Z
1000	0.285	0.276	0.294	0.248	0.240	0.512	0.339	0.323	0.338	0.314	0.300	0.385
2000	0.285	0.261	0.285	0.256	0.234	0.510	0.347	0.314	0.339	0.321	0.294	0.386
5000	0.289	0.266	0.290	0.255	0.235	0.511	0.342	0.316	0.342	0.318	0.294	0.387
10000	0.287	0.267	0.288	0.254	0.237	0.510	0.339	0.318	0.343	0.318	0.296	0.387

In this example, the average standard deviations of *X*, *Y*, and *Z* are relatively close, suggesting that the theoretically optimal prior weights should be 0.25, 0.25, and 0.5, respectively. However, the CRITIC-Cor method consistently provides weights near 0.33, 0.33, and 0.33 for each indicator, which deviates markedly from the expected values. This misalignment is primarily due to the Pearson correlation coefficient used in the CRITIC-Cor method, which fails to capture non-linear relationships among the variables. Consequently, it incorrectly assumes that *X*, *Y*, and *Z* are independent, leading to an equal initial weight distribution of 0.33 for each.

Moreover, since the average standard deviations are nearly identical, this further biases the CRITIC-Cor method towards equal weights of 0.33 across all indicators. While the CRITID-Dcor method does not achieve weights as close to the ideal 0.25, 0.25, and 0.5 as might be desired, it nevertheless adheres more closely to the required weight relationships and thus approaches optimal prior weight distribution more closely than the CRITIC-Cor method. Therefore, despite its imperfections, the CRITID-Dcor method represents a more accurate and reliable approach in scenarios where variable relationships are complex and potentially non-linear. In Example 3.4, the MSE for CRITIC-Cor stands at 1.447%, while CRITID-Dcor shows an improved performance with an MSE of 0.775%.

### Sensitivity analysis

#### Example 4.1

 We conducted a simulation study involving four criteria: A, B, C, and D. Each criterion was modeled to follow the same statistical distribution, and we assessed their robust performance across four different types of distributions: standard normal distribution, exponential distribution with a parameter of 1, Poisson distribution with a parameter of 1, and uniform distribution ranging from − 10 to 10.

To evaluate the robustness of the criteria against outliers, we introduced one outlier to each criterion, calculated as the mean of the distribution plus four standard deviations of that distribution. This simulation was repeated 500 times to ensure statistical reliability. The primary variable of interest in our analysis was the average weight change rate for each criterion, evaluated under varying distributions. This metric was calculated for both the CRITIC-Cor method and the CRITID-Dcor method, enabling a comparative analysis of their responses to outliers.

**Figure 12:**
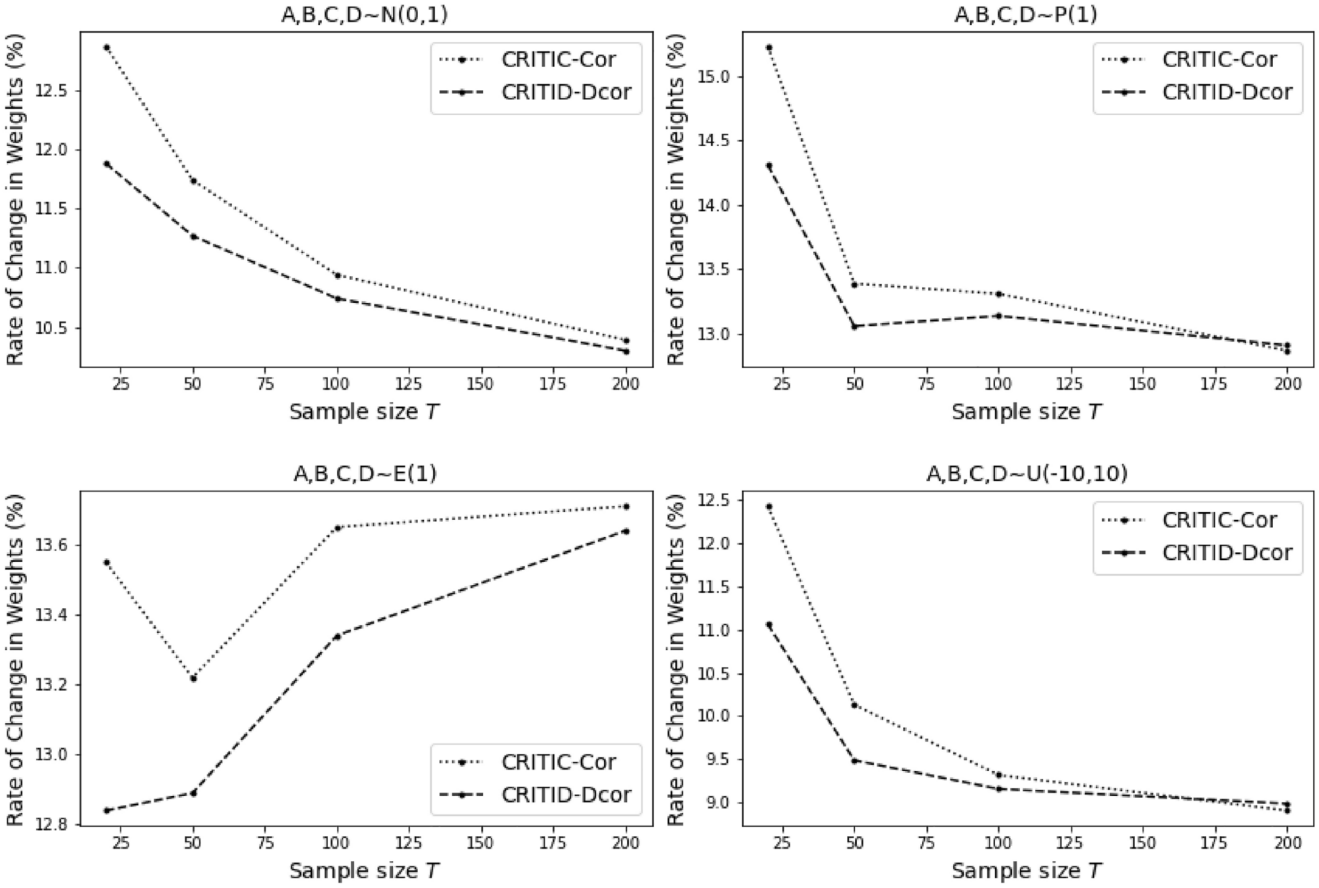
Sensitivity analysis of the weight change rate in Example 4.1.

The results of these simulations (Table 11 and Fig. 12) provide insights into how each method adapts to outliers and changes in distribution characteristics, highlighting differences in robustness between the CRITIC-Cor and CRITID-Dcor methods.

**Table 11:** Sensitivity analysis of weight change rate for CRITIC-Cor and CRITID-Dcor in response to outliers in Example 4.1.

	A,B,C,D~N(0,1)		A,B,C,D~P(1)		A,B,C,D~E(1)		A,B,C,D~U(-10,10)	
	CRITIC-Cor (%)	CRITID-Dcor (%)	CRITIC-Cor (%)	CRITID-Dcor (%)	CRITIC-Cor (%)	CRITID-Dcor (%)	CRITIC-Cor (%)	CRITID-Dcor (%)
T = 20	12.87	11.88	15.23	14.31	13.55	12.84	12.42	11.06
T = 50	11.74	11.27	13.39	13.06	13.22	12.89	10.13	9.49
T=100	10.94	10.74	13.31	13.14	13.65	13.34	9.32	9.16
T=200	10.39	10.3	12.87	12.91	13.71	13.64	8.91	8.99

As the sample size increases, the average weight change rate for both methods generally decreases. This trend occurs because the fixed outliers become diluted as the sample size grows, reducing their impact on the overall dataset.

Overall, the CRITID-Dcor method exhibits a lower average weight change rate compared to the initial CRITIC-Cor method across various distributions, with this difference being particularly pronounced in smaller sample sizes. When the sample size is large, the average change rates of the two methods converge, indicating similar robustness. Nevertheless, the CRITID-Dcor method consistently demonstrates improved robust performance over the CRITIC-Cor method, especially in scenarios with smaller samples.

## Real data analysis

### Parkinson’s disease data

This example focuses on the objective weighting of unique voice characteristics of patients with Parkinson’s disease^29^ to enhance the detection of the disease. The features considered in this analysis include:

- MDVP:Fo(Hz) - Average vocal fundamental frequency;
- MDVP:Fhi(Hz) - Maximum vocal fundamental frequency;
- Shimmer:APQ5 - Five-point amplitude perturbation quotient.

Among them, the Pearson correlation coefficient and distance correlation coefficient matrix of the features MDVP:Fo(Hz), MDVP:Fhi(Hz) and Shimmer:APQ5 are as follows in Tables 12 and 13.

**Table 12:** Pearson correlation coefficient matrix for Parkinson’s disease data.

	MDVP:Fo(Hz)	MDVP:Fhi(Hz)	Shimmer:APQ5
MDVP:Fo(Hz)	1.0000	0.4009	-0.0707
MDVP:Fhi(Hz)	0.4009	1.0000	-0.0099
Shimmer:APQ5	-0.0707	-0.0099	1.0000

**Table 13:** Distance correlation coefficient matrix for Parkinson’s disease data.

	MDVP:Fo(Hz)	MDVP:Fhi(Hz)	Shimmer:APQ5
MDVP:Fo(Hz)	1.0000	0.7225	0.1773
MDVP:Fhi(Hz)	0.7225	1.0000	0.1527
Shimmer:APQ5	0.1773	0.1527	1.0000

**Figure 13:**
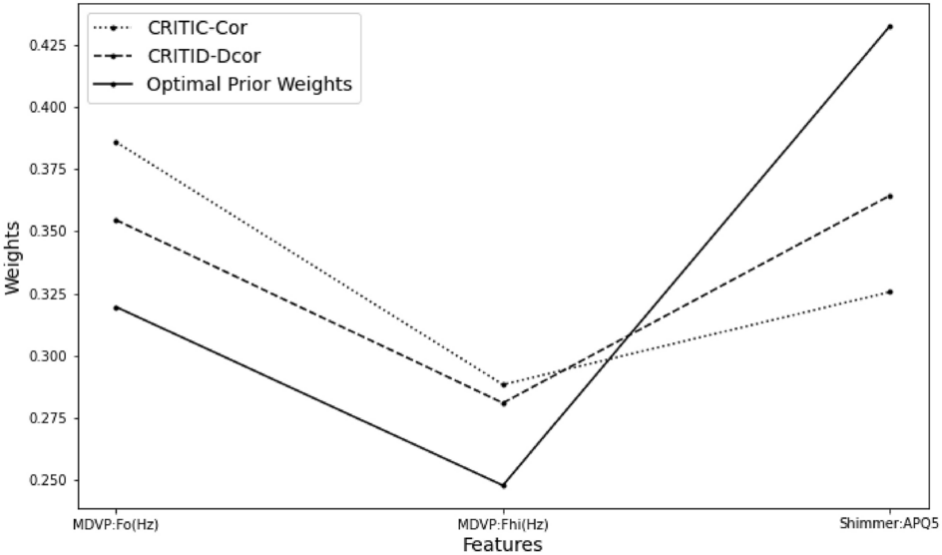
Detailed comparison and analysis results for Parkinson’s disease data.

According to the distance correlation coefficient matrix above, we can see that the correlation between MDVP:Fo(Hz) and MDVP:Fhi(Hz) is very strong, the correlation between MDVP:Fo(Hz) and Shimmer:APQ5 is weak, and the correlation between MDVP:Fhi(Hz) and Shimmer:APQ5 is also weak. According to the optimal prior weights we proposed, the initial weights of the three criteria should be 0.25, 0.25, and 0.5 without considering the standard deviation. After considering the standard deviations of the three, the optimal prior weights of the three, the CRITIC-Cor method weight, and the CRITID-Dcor method weight are calculated as shown in the Table 14 and Fig. 13 below.

**Table 14:** Comparative analysis of weights calculated by CRITIC-Cor and CRITID-Dcor methods against optimal prior weights for Parkinson’s disease data.

	MDVP:Fo(Hz)	MDVP:Fhi(Hz)	Shimmer:APQ5
Standard deviation	0.2409	0.1867	0.1631
Optimal prior weight	0.3196	0.2477	0.4327
CRITIC-Cor weight	0.3860	0.2883	0.3256
CRITID-Dcor weight	0.3546	0.2809	0.3644

Based on the proposed optimal prior weights, it is evident that Shimmer:APQ5 should receive the highest weight. However, the CRITIC-Cor method assigns the maximum weight to MDVP:Fo(Hz), which renders this approach unreasonable. Conversely, the CRITID-Dcor method calculates weights that are more closely aligned with the optimal prior weights. The rationale for this discrepancy is that the distance correlation coefficient between MDVP:Fo(Hz) and MDVP:Fhi(Hz) is significantly higher than the Pearson correlation coefficient between the two, indicating a strong non-linear correlation that the Pearson correlation cannot capture.

In practical applications, using the initial CRITIC method would likely result in an excessive weight assigned to MDVP:Fo(Hz) and insufficient weight to Shimmer:APQ5, leading to a potentially unreasonable allocation of weights. Utilizing the CRITID-Dcor method, on the other hand, allows for a more balanced distribution of weights. Of course, in real-world scenarios, in addition to objective weights, subjective weights must also be considered. This requires subjective judgment by professional clinicians to adjust weights appropriately based on clinical insights and expertise.

### Smart education evaluation data

This case study concentrates on the objective weighting of fifteen indicators derived from the data collected across various educational stages in different districts of a city in the western part of China. These indicators are designed to reflect diverse aspects of educational infrastructure and IT integration, which include:

- Indicator 1: Number of schools;
- Indicator 2: Student population;
- Indicator 3: Number of teachers;
- Indicator 4: Number of school classes;
- Indicator 5: Number of classrooms for teaching;
- Indicator 6: Number of computer classrooms for students;
- Indicator 7: Number of information technology teachers;
- Indicator 8: Number of full-time information technology teachers;
- Indicator 9: Number of teachers participating in information technology training this year;
- Indicator 10: Number of information technology school teachers involved in training;
- Indicator 11: Number of non-information technology school teachers involved in training;
- Indicator 12: Number of schools with an information management officer;
- Indicator 13: Number of schools with a Chief Information Officer;
- Indicator 14: Total number of computers;
- Indicator 15: Number of desktop computers.

Table 15 presents partial data for some of these indicators.

**Table 15:** Detailed distribution of educational resources by district.

District	Number of schools	Number of students	Number of teachers	Number of school classes	Number of teaching classrooms
A District	47	82,061	5,185	1,839	2,208
B District	51	87,316	6,567	1,888	2,631
C District	53	50,828	7,264	1,477	3,400
D District	48	42,679	3,121	989	1,126
E District	54	62,027	5,134	1,440	1,894

By employing the CRITIC-Cor and CRITID-Dcor methods, we determined the weights of the specified indicators﻿, as presented in Table 16. Objective weighting of this dataset facilitates the assessment of informatization levels within specific administrative districts of the city, thereby enhancing the strategic allocation of educational resources.

To effectively illustrate the robustness of these methods, particularly in response to data anomalies, we introduce an outlier scenario. This involves inserting a value twice as large as the maximum recorded value for each indicator and observing the resultant impacts on the weighting outcomes of both the CRITIC-Cor and CRITID-Dcor methods. Such a comparison underscores the sensitivity of these methods to outliers and their capacity to manage extreme variations in data.

**Figure 14:**
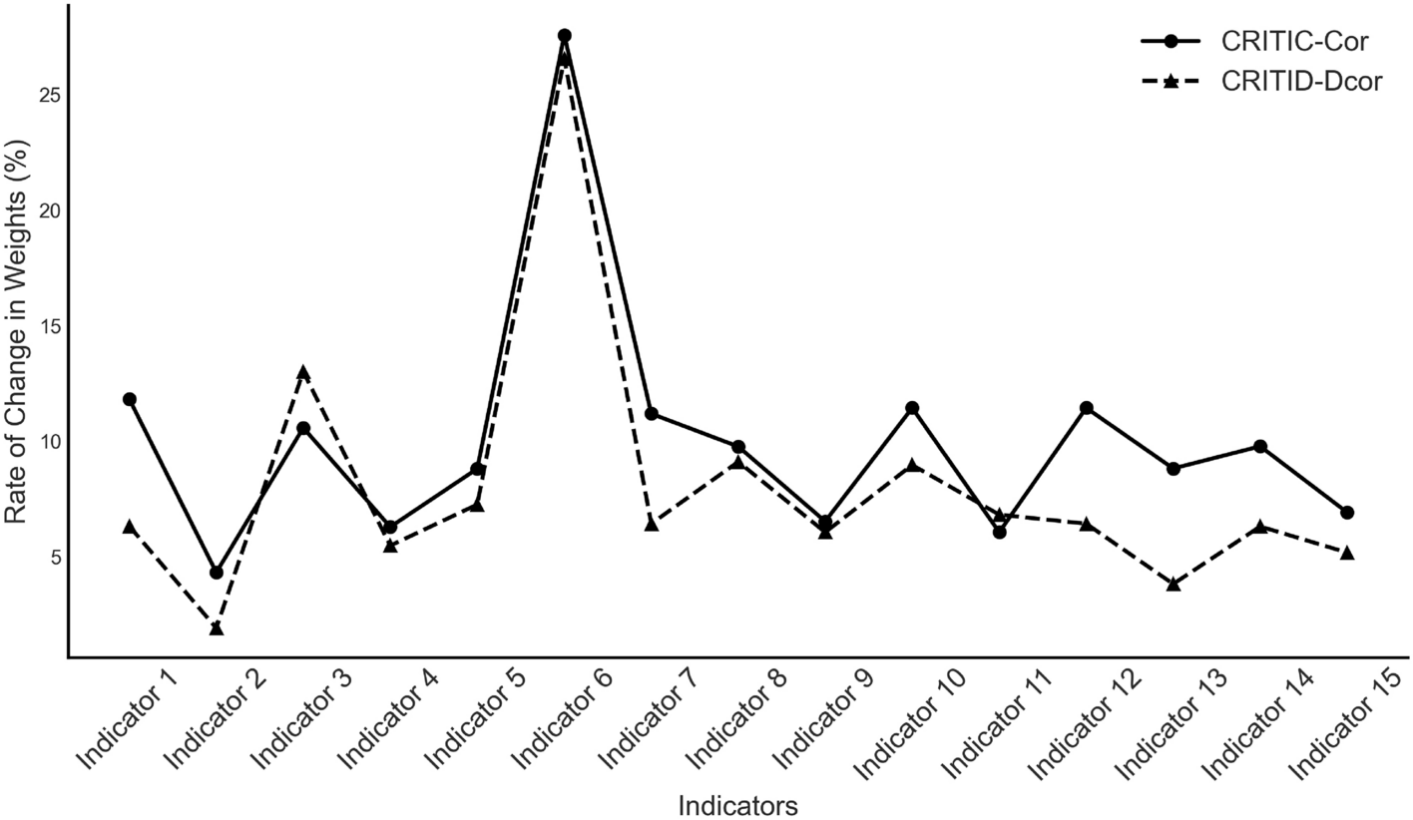
Sensitivity analysis of educational evaluation metrics in response to outliers.

We introduced two outliers for each indicator, with the results displayed in Table 16 and Fig. 14. Post-introduction, both the CRITIC-Cor and the CRITID-Dcor methods showed increased variability in weight changes. The CRITID-Dcor method generally exhibited lower change rates than the original method for most indicators. On average, the CRITID-Dcor method experienced a mean weight change rate of 7.97%, compared to the 10.09% observed with the CRITIC-Cor. This reinforces the CRITID-Dcor method’s stability and reliability in handling datasets with significant variability or outliers.

**Table 16:** Comparative and sensitivity analysis of CRITIC-Cor and CRITID-Dcor weights in response to outliers for the educational evaluation data.

Indicator	CRITIC-Cor weight	CRITID-Dcor weight	CRITIC-Cor weight change rate (%)	CRITID-Dcor weight change rate (%)
Indicator 1	0.060	0.060	11.82	6.32
Indicator 2	0.045	0.047	4.32	1.91
Indicator 3	0.049	0.055	10.58	13.01
Indicator 4	0.042	0.044	6.29	5.48
Indicator 5	0.055	0.057	8.81	7.26
Indicator 6	0.064	0.059	27.58	26.56
Indicator 7	0.065	0.062	11.19	6.43
Indicator 8	0.061	0.065	9.77	9.11
Indicator 9	0.083	0.082	6.53	6.06
Indicator 10	0.077	0.076	11.44	8.97
Indicator 11	0.085	0.081	6.06	6.82
Indicator 12	0.065	0.066	11.44	6.43
Indicator 13	0.153	0.142	8.82	3.82
Indicator 14	0.046	0.050	9.78	6.31
Indicator 15	0.051	0.055	6.92	5.18

## Summary

With the continuous development of the objective weighting method, it has been increasingly applied in various fields such as finance, education, and environmental protection. Generally, determining the weight of criteria involves considering both linear and non-linear correlations between criteria.

This work integrates distance correlation and other independence testing methods into the CRITIC framework, resulting in the development of a new objective weighting method named CRITID. This enhancement leverages advanced statistical tools to refine the accuracy and reliability of weight assessments in multi-criteria decision-making processes. In the section on simulated numerical experiments, we demonstrate through simulated data samples and repeated experiments that the CRITID-Dcor method is more reasonable and robust than the original CRITIC method. In the practical examples section, we demonstrate the efficiency and robustness of the CRITID-Dcor method through two specific examples.

Although the CRITID-Dcor method offers greater reasonableness and robustness compared to the original CRITIC method, it is not without drawbacks. Notable limitations include its high time complexity, which could hinder its application in more dynamic settings. The time complexity of the proposed CRITID method depends on the independence test methods employed. Utilizing the distance correlation method, CRITID-Dcor, results in an overall time complexity of $O(n^2)$, where *n* represents the sample size. Further research is required to enhance the computational efficiency of this algorithm.

The proposed CRITID method can be integrated with fuzzy set theory to address challenges related to uncertain and imprecise data, as discussed in^12,30,31^. Further studies are necessary to assess the method’s effectiveness in managing complex and diverse nonlinear scenarios, such as those encountered in the Productive Economic Endeavors Distribution Problem^32^, and in the teaching quality evaluation problem^33^.

## Data Availability

The datasets used and/or analysed during the current study available from the corresponding author on reasonable request.
